# Tumor-associated long non-coding RNAs show variable expression across diffuse gliomas and effect on cell growth upon silencing in glioblastoma

**DOI:** 10.1038/s41598-025-99984-9

**Published:** 2025-05-09

**Authors:** Joonas Uusi-Mäkelä, Maria Kauppinen, Janne Seppälä, Serafiina Jaatinen, Birgitta Ryback, Tommi Rantapero, Alejandra Rodriguez-Martinez, Matti Nykter, Kirsi J. Rautajoki

**Affiliations:** 1 Prostate Cancer Research Center, Faculty of Medicine and Health Technology, Tampere University, Tampere, Finland; 2 IT Management, Helsinki University Hospital, Helsinki, Finland; 3 Department of Cancer Biology, Dana-Farber Cancer Institute, Boston, MA USA

**Keywords:** CNS cancer, Non-coding RNAs

## Abstract

Long noncoding RNAs (lncRNAs) have been recently recognized as critical components of cancer biology linked to oncogenic processes. Certain lncRNAs are known to act as oncogenes, and the disease-specific expression of many lncRNAs makes them informative biomarkers. We identified 22 uncharacterized lncRNAs from RNA-seq data of 169 glioblastoma (GBM) tumor samples sequenced by The Cancer Genome Atlas (TCGA) consortium and studied their expression in TCGA diffuse glioma cohort including also IDH-mutant astrocytomas and oligodendrogliomas as well as in normal brain samples from the Genotype-Tissue Expression cohort. All of the 22 lncRNAs were clearly upregulated in diffuse gliomas samples compared to the normal brain. Interestingly, 20 (91%) of these lncRNAs had significant expression differences between tumor grades and/or entities, and 14 (64%) were associated with overall patient survival. All 22 lncRNAs were expressed in at least one of the studied GBM cell lines and 10 (45%) were expressed in all four. When six of the lncRNAs were silenced in the SNB19 GBM cell line, the knock-down was associated with reduced growth and colony formation for three lncRNAs: *TCONS_l2_00001282*, *lnc-GBMT-6*, and *lnc-NBN-1*. In conclusion, the studied lncRNAs are associated with survival in patients with diffuse glioma and have functional relevance in GBM.

## Introduction

The most common primary brain cancer, glioblastoma (GBM), is an aggressive, treatment-resistant, and fatal disease with an average survival of only 17 months^1^. The standard treatment for GBM is a combination of radiotherapy and the alkylating agent temozolomide (TMZ)^2^. Other adult diffuse gliomas are IDH-mutated (IDHmut) astrocytomas and oligodendrogliomas, both of which harbor hotspot *IDH* mutations^3^. IDHmut astrocytomas are found as grade 2–4 tumors. Oligodendrogliomas represent grades 2–3 and feature the codeletion of chromosome arms 1p and 19q which is used in diagnostics.

Protein-coding genes represent only approximately 2% of the whole human genome; however, more than 70% of the genome produces transcripts^4^. Not all these transcripts are functional but more than half of them are currently annotated as genes that produce noncoding RNAs (ncRNAs)^5^. These ncRNAs are divided into two categories based on length: small ncRNAs (sRNAs), which are under 200 bp and long ncRNAs (lncRNAs), which are over 200 bp. The majority of ncRNAs are lncRNAs that are processed in similar ways as mRNAs in cells^6^. There are four main mechanisms by which lncRNAs function. The most studied function is the guide function, where lncRNAs can bring two different macromolecules in close proximity to each other. Some notable examples are polycomb repressive complex 2, which requires RNA binding for correct chromatin localization in human pluripotent stem cells, and the lncRNA SWINGN, which is needed for SWI/SNF complexes to activate specific promoters^7,8^. In addition to guiding, lncRNAs can act as scaffolds to regulate certain processes, such as chromatin looping between enhancers and promoters^9^. A third possible mechanism involves binding molecules for targeted inhibition to function as sponges. One example of these sponges is the lncRNA called Cyrano which can act as a binding site for several miR-7 molecules, inhibiting the function of miR-7^10^. Finally, although counterintuitive, lncRNAs can be translated to peptides with regulatory roles; *e.g.,* mTORC1 is regulated by the peptide encoded by a lncRNA^11^.

When lncRNA expression in the brain is compared with that in other tissues, approximately 40% of the lncRNAs have brain-specific expression patterns^12^. This is also interesting compared with protein-coding genes, as fewer unique proteins (with respect to all genes encoding proteins) are expressed in the brain. One explanation for this high brain-specific expression pattern of lncRNAs could be the dynamic regulation of lncRNAs during neuronal development and the fact that different parts of the brain tend to have their own specific lncRNAs^13,14^.

In GBMs, there are at least 13 reported driver mutations located in ncRNAs, although the functions of these ncRNAs are not clear^15^. However, deregulation of lncRNAs has been reported in several studies, and at least 107 lncRNAs have been reported in GBMs^16^. Among these 107 lncRNAs, most have been reported in only a single study and have not been studied further^16^. Four lncRNAs are well-described: *HOTAIRM1, NEAT1, MEG3,* and *MALAT1*^17–19^. All of the well-described cases function through sponge mechanisms, such as regulating miRNAs and mRNAs. However, out of 107 deregulated candidates, only 21 have been studied in more than one publication, making it clear that more research is needed on this topic.

The identification of novel ncRNAs from deep sequencing data enables the identification of more relevant regulators for normal and pathological conditions, including GBMs and other diffuse gliomas. Different tools have been developed for this purpose, with variable features such as different input and output formats and the ability to detect known and novel ncRNAs^20^. Here, we identified several non-annotated lncRNAs in GBMs using an in-house developed method. We further analyzed and validated selected transcripts to determine their functional roles in GBM cells, variable expression patterns in diffuse gliomas, and associations with patient survival.

## Results

### Detection of ncRNAs from GBM samples allows the identification of novel lncRNAs

**Fig. 1:**
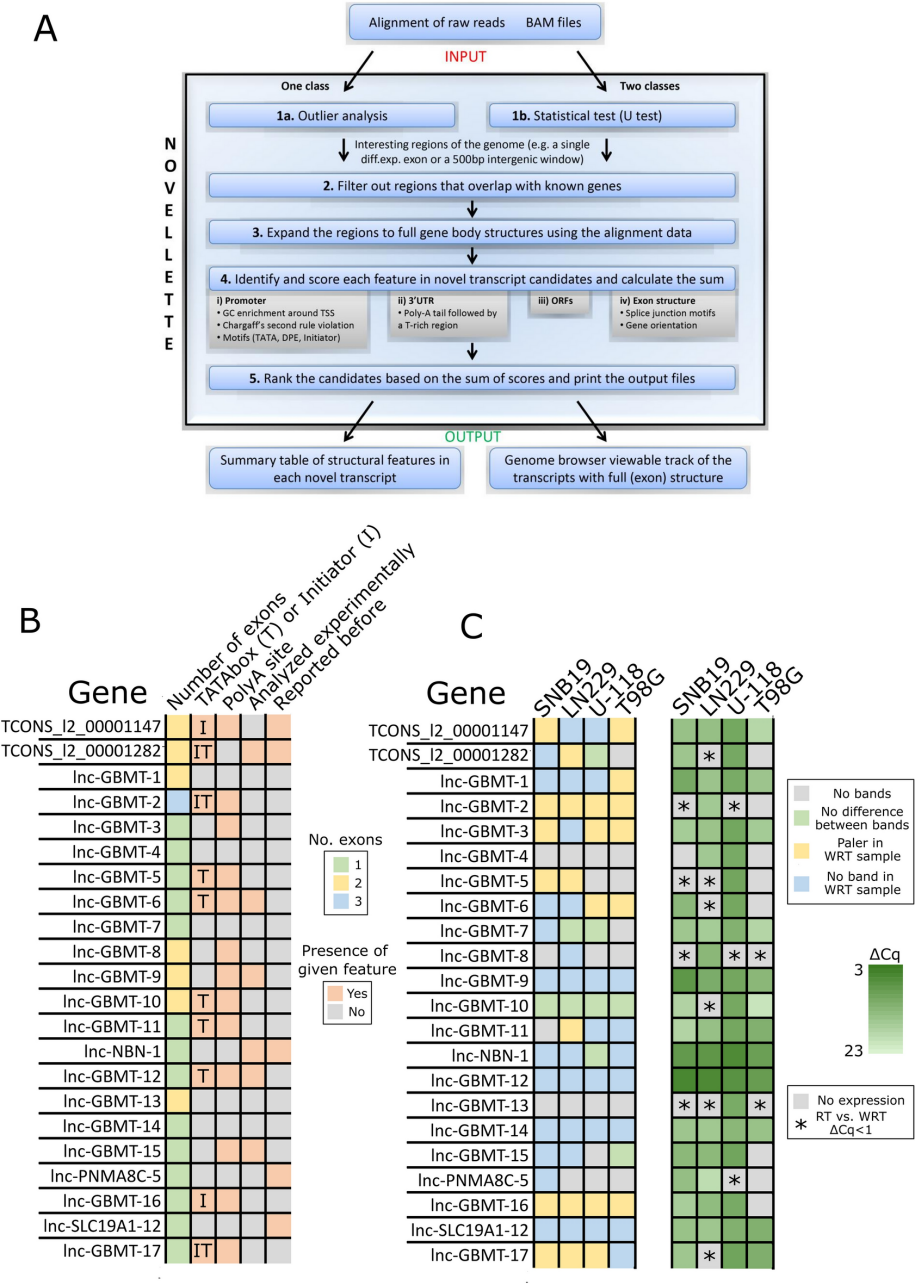
Novel transcripts harboring typical gene body structures detected in TCGA GBM RNA-seq data. (**a**) Workflow of putative lncRNA detection. The input is either an alignment of raw reads or BAM files followed by outlier analysis or a statistical test. Regions overlapping with known genes are filtered out, and the resulting regions are expanded to full gene body structures. Hits are scored based on different features and ranked. (**b**) Heatmap visualization showing gene body structure information for the 22 novel lncRNAs as well as whether they were analyzed functionally in this study or previously reported in either the UCSC genome browser or LNCipedia. (**c**) Heatmap visualization summarizing the expression of novel lncRNAs in GBM cell lines. On the left side: lncRNAs were considered to be expressed when a band was detected after reverse transcription PCR (RT-PCR), and there was no band when RT-PCR was performed without reverse transcriptase before PCR (without reverse transcriptase, WRT). Additionally, when the band was clearly paler in WRT than in RT-PCR, the lncRNA was considered to be expressed. The idea was to separate RNA-derived signals from signals originating from possible DNA contamination. On the right side: quantitative RT-PCR (qPCR) from all the cell lines. Heatmap color represents ΔCq values of lncRNAs when compared to GAPDH values from the same cell line. lncRNAs were considered not to be expressed if there was no signal from the sample or if ΔCq < 1 between RT-sample and WRT-sample (marked with an asterisk).

We developed and utilized an in-house method for identifying novel ncRNAs from The Cancer Genome Atlas (TCGA) GBM RNA-sequencing (RNA-seq) dataset^21^. The method scans the whole genome in 500 bp sliding windows and finds interesting regions (*e.g.,* windows with variable expression within the dataset) (Fig. 1A). After that, the regions overlapping known genes are filtered out to leave only novel transcripts, which are then expanded to full gene structures, when applicable, using the alignment data. These novel transcript candidates are then scored based on the presence of the typical promoter region sequences, 3’UTR, open reading frame(s) (ORFs), and exonic structure (see Methods for more details). When 169 GBM samples, including mainly IDH-wildtype GBMs and a subpopulation of grade 4 IDHmut astrocytomas (diagnosed previously as GBMs), were used as inputs, 53 intergenic putative lncRNAs were obtained as outputs.

The aligned RNA sequencing reads for putative novel lncRNAs were then analyzed with Interactive Genomics Viewer (IGV) to obtain a better understanding of the lncRNA boundaries, possible exon–intron structures, and the presence of typical gene motifs and to detect protein-coding and other nearby genes (Supplementary Fig. 1, Supplementary Table 1), which reduced the number of possible candidates to 22. Each of these 22 lncRNAs had at least one exon, 10 (45%) had an initiator or TATA box in the expected location at the promoter region, and 13 (59%) had a poly(A) tail site at the 3’ end (Fig. 1B, Supplementary Table 1). Next, PCR was used to evaluate whether the 22 lncRNAs were expressed in four GBM cell lines (SNB19, LN229, U-118, and T98G). A total of 11 lncRNAs (50%) were present in all the cell lines (Fig. 1C, Supplementary Fig. 2, Supplementary Table 1), and all but three (14%) in at least one cell line. Samples for which reverse transcription was performed without presence of the reverse transcriptase were used as controls for contaminating DNA. This analysis was repeated with quantitative PCR (qPCR), and the results were similar (Fig. 1C, Supplementary Fig. 3A, Supplementary Table 1). With qPCR, we were able to detect each lncRNA in at least one cell line and eight (36%) lncRNAs in all the cell lines used (Fig. 1C, Supplementary Table 1). These results show that these novel lncRNAs are expressed in GBM cells themselves.

To study the novelty of lncRNAs, we analyzed the genomic annotations in the UCSC genome browser TUCP transcripts track^22^. Similarly, we searched LNCipedia to find if these lncRNAs are reported there^23^. We found five matches for our lncRNAs (Fig. 1B): *TCONS_l2_00001147, TCONS_l2_00001282, lnc-NBN-1, lnc-PNMA8C-5*, and *lnc-SLC19A1-12* have been reported before.

### lncRNAs exhibit differential expression and are associated with survival in diffuse gliomas

**Fig. 2:**
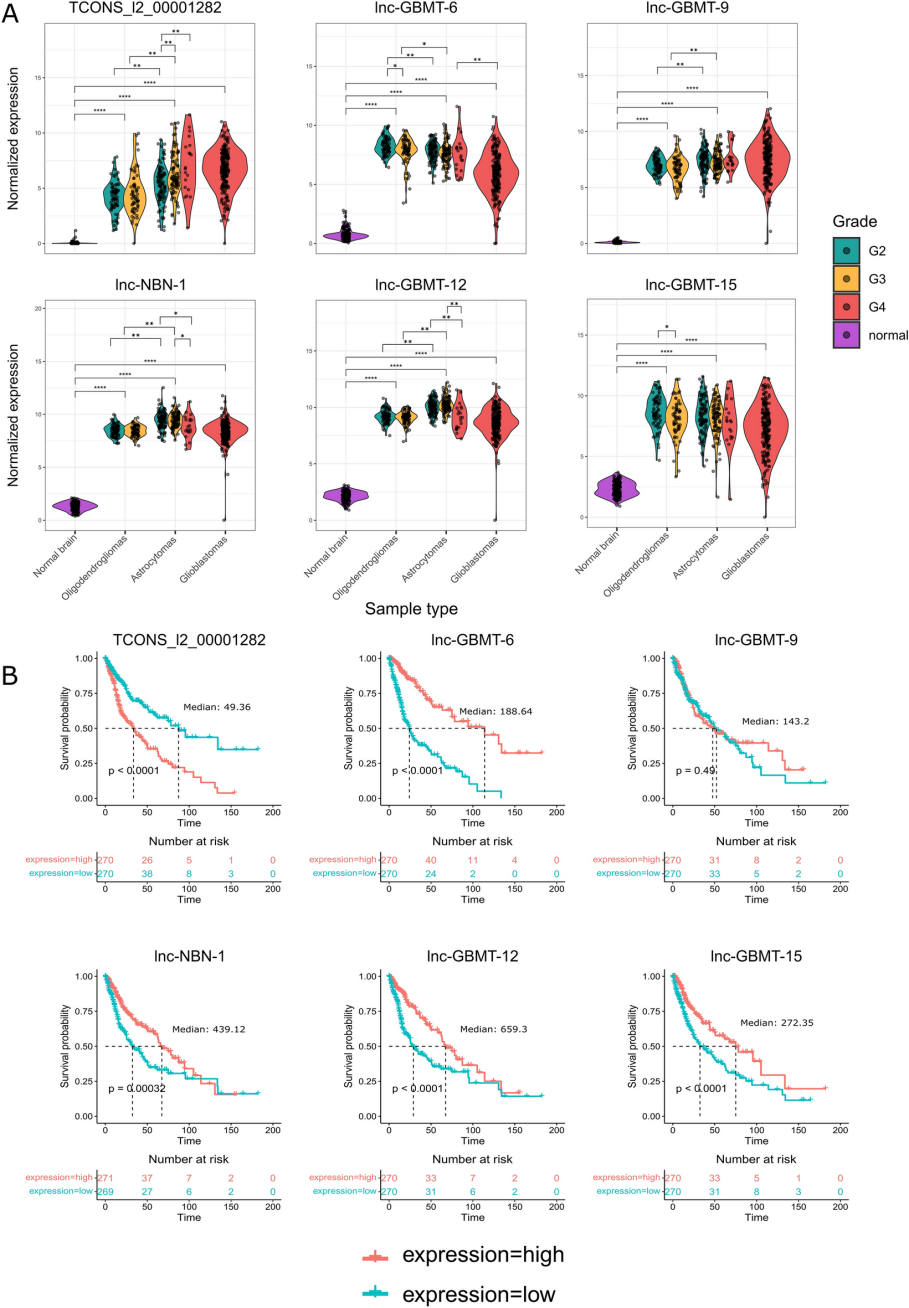
Novel lncRNAs are expressed on GBMs and IDH-mutant gliomas and harbor expression differences across and within different tumor entities. (**a**) Expression patterns of six different lncRNAs in grade 2–3 oligodendrogliomas, grade 2–4 astrocytomas, and grade 4 GBMs. and normal brain. Expression differences were calculated between different grades within one tumor entity or between tumor entities representing the same tumor grade or between normal brain and tumor entities. *p < 0.05, **p < 0.01, ****p < 0.0001, Wilcoxon rank-sum test. (**b**) Kaplan–Meier visualizations of the survival analysis in the whole diffuse glioma cohort. The mean expression level was used as a cutoff for low/high lncRNA expression. All the lncRNAs except *lnc-GBMT-9* were significantly associated with overall patient survival. Statistical significance was calculated with a log-rank test.

We then studied the expression of these 22 lncRNAs in the TCGA diffuse glioma cohort. The cohort included IDHmut astrocytomas and oligodendrogliomas in addition to GBMs, and the tumor grades ranged from 2 to 4 (Fig. 2A, Supplementary Fig. 4A, Supplementary Table 1). We also obtained gene expression data of normal brain samples from The Genotype-Tissue Expression (GTEx) portal. All of the studied lncRNAs had lower expression in the normal brain when compared to diffuse gliomas, showing that these lncRNAs are mostly expressed in tumors. (Fig. 2A, Supplementary Fig. 4A, Supplementary Table 1). In 16/22 (72%) of the lncRNAs, there were expression differences between different grades within IDHmut astrocytomas and/or oligodendrogliomas (Fig. 2A, Supplementary Fig. 4A, Supplementary Table 1). Nine of these lncRNAs exhibited decreasing expression with increasing tumor grade in oligodendrogliomas. In IDHmut astrocytomas, three lncRNAs were upregulated and nine were downregulated with increasing tumor grade. Furthermore, 18/22 (82%) lncRNAs presented expression differences between the grades 2-3 in different tumor types (*e.g.,* between grade 3 IDHmut astrocytoma and grade 3 oligodendroglioma). Notably, some lncRNAs, such as *TCONS_l2_00001282,* were significantly different between both tumor entities and tumor grades. When only grade 2 tumors were examined, 12 lncRNAs were more highly expressed in IDHmut astrocytomas than in oligodendrogliomas, and five were more highly expressed in oligodendrogliomas. In grade 3 tumors, 12 lncRNAs presented higher and five presented lower expression in IDHmut astrocytomas than in oligodendrogliomas. Finally, when the most aggressive tumors, namely, grade 4 IDHmut astrocytomas and GBMs, were compared, 2/22 (9%) of the lncRNAs had lower expression in GBMs (including *lnc-GBMT-6*), and 2/22 (9%) had higher expression in GBMs (Fig. 2A, Supplementary Fig. 4A, Supplementary Table 1). To conclude, these 22 lncRNAs have very diverse expression profiles, representing differences between tumor grades and entities and expression of these lncRNAs in the normal brain is clearly lower than in diffuse gliomas.

Next, we studied whether the expression of these novel lncRNAs is associated with overall patient survival (Fig. 2B, Supplementary Fig. 5). We studied survival in patients with different tumor types and in the whole diffuse glioma cohort. When all the tumor entities were combined, expression 14/22 (64%) of lncRNAs was associated with survival (p < 0.05, log-rank test), when patients were divided based on median lncRNA expression (Fig. 2B, Supplementary Fig. 5A). Eleven lncRNAs were positively associated with survival, and three were negatively associated with survival (Fig. 2B, Supplementary Fig. 5A). Two of the negatively associated lncRNAs (*lnc-GBMT-2* and *TCONS_l2_00001282*) showed higher expression in IDHmut astrocytomas than oligodendrogliomas and upregulation with an increasing grade within IDHmut astrocytomas. The third negatively associated lncRNA, *lnc-GBMT-11*, was more highly expressed in GBM than IDH-mutant tumors. None of these was associated with patient prognosis within individual diffuse glioma subtypes. Furthermore, out of 11 lncRNAs that were positively associated with survival, all but one showed decreased expression with increasing tumor grade within IDHmut astrocytoma and/or oligodendroglioma and all but two were differentially expressed between diffuse glioma subtypes representing the same tumor grade. Four of these were similarly associated with longer survival within individual diffuse glioma subtypes: *lnc-GBMT-7, lnc-GBMT-10*, and *lnc-PNMA8C-5* within GBM and *lnc-SLC19A1-12* within GBM and oligodendroglioma.

There were two lncRNAs whose expression differed significantly between grade 4 IDHmut astrocytomas and GBMs, and survival was affected accordingly. LncRNA *lnc-GBMT-11* was expressed at higher levels in GBM than in grade 4 IDHmut astrocytoma, and higher *lnc-GBMT-11* expression was associated with worse survival in the diffuse glioma cohort. LncRNA *lnc-GBMT-6* was expressed at lower levels in GBM than in grade 4 IDHmut astrocytoma and also at lower levels in IDHmut astrocytomas than oligodendrogliomas. As expected, its lower expression was associated with worse survival in the diffuse glioma cohort (Fig. 2B, Supplementary Figs. 4A and 5A, Supplementary Table 1).

In the oligodendroglioma cohort, 4/22 (18%) lncRNAs were associated with better patient survival (Supplementary Fig. 5C), of which two (*lnc-GBMT-5* and *lnc-GBMT-8*) were also expressed at lower levels in high-grade oligodendroglioma tumors and only one (*lnc-SLC19A1-12*) was significantly associated with patient survival also in the whole diffuse glioma cohort (Supplementary Figs. 4A and 5C, Supplementary Table 1). In IDHmut astrocytomas 2/22 (9%) (*lnc-GBMT-9* and *lnc-GBMT-17*) lncRNAs were associated with better survival, and one (*lnc-GBMT-17*) also presented lower expression in higher-grade IDHmut astrocytomas (Supplementary Figs. 4A and 5B, Supplementary Table 1). Finally, 4/22 (18%) lncRNAs were associated with better survival in the GBM cohort, which represent only one tumor grade (Supplementary Figs. 4A and 5D, Supplementary Table 1).

Our results revealed a stronger association with survival when all the tumor entities were pooled for the analysis. This is expected, as 12 out of 14 lncRNAs associated with survival in the whole diffuse glioma cohort showed expression differences between tumor entities and 12 lncRNAs between grades of one tumor entity. In adult diffuse gliomas, GBMs are associated with the shortest survival and oligodendrogliomas with the longest survival^24^, so expression differences between subtypes are likely to influence survival associations. Interestingly, although we originally detected these lncRNAs in cohort with mostly GBM samples, they were also expressed in low-grade IDH-mutant tumors, and some of them were associated with survival in patients with IDH-mutant tumors. These findings suggest that the lncRNAs identified in this study also play roles in IDH-mutant diffuse gliomas in addition to IDH-wildtype GBM.

### Growth and colony formation are reduced in the SNB19 cell line after the silencing of three lncRNAs

We then wanted to study whether our lncRNAs have functional effects when silenced. Based on their expression in cell lines and tumor samples as well as their survival associations, six lncRNAs were chosen for functional experimentation: *TCONS_l2_00001282*, *lnc-GBMT-6*, *lnc-GBMT-9, lnc-NBN-1, lnc-GBMT-12* and *lnc-GBMT-15*. All these lncRNAs were expressed in the SNB19 cell line, which was used for the analysis.

**Fig. 3:**
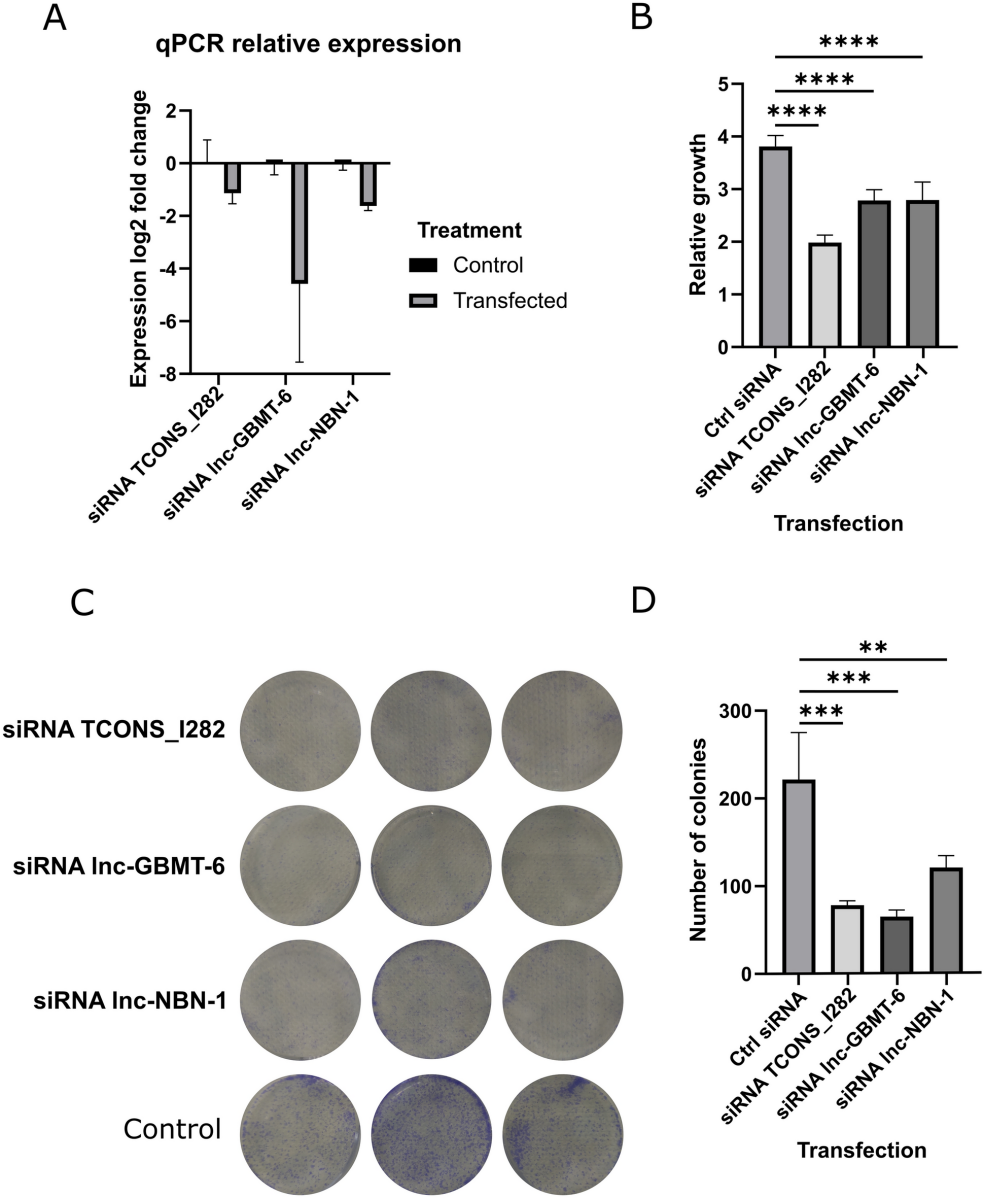
Silencing of selected lncRNAs in the SNB19 GBM cell line leads to reduced growth and colony formation. (**a**) Relative expression levels of *TCONS_l2_00001282, lnc-GBMT-6*, and *lnc-NBN-1* after targeted or control siRNA transfection in the SNB19 cell line. All of the lncRNAs presented reduced expression after siRNA transfection. The results show the mean expression difference together with the standard deviation from measurements representing three separate biological experiments (separate wells on a well-plate), each with three technical replicates (separate measurements from one well). (**b**) Alamar Blue assay showing reduced cell growth after the silencing of lncRNAs *TCONS_l2_00001282, lnc-GBMT-6,* and *lnc-NBN-1*. The figure shows the mean relative growth from six biological replicates (separate wells on a well-plate) with the standard deviation after five days in comparison with that on day zero. ****p < 0.0001, one-way ANOVA. From each biological replicate, six technical replicates (separate measurements from one well) were taken for the Alamar Blue assay. The results for similar, significantly reduced cell growth were acquired in two separate experiments in the case of *TCONS_l2_00001282* and in three separate experiments for *lnc-GBMT-6* and *lnc-NBN-1*. (**c**) Representative images of cell culture dishes after the clonogenic assay for *TCONS_l2_00001282, lnc-GBMT-6*, and *lnc-NBN-1* lncRNAs that had growth effects in the Alamar Blue assay. d) Cell colony formation was reduced after the silencing of *TCONS_l2_00001282, lnc-GBMT-6, or lnc-NBN-1* lncRNAs in the SNB19 cell line. The figure shows the mean number of colonies from three biological replicates (separate wells on a well-plate) together with the standard deviation after transfection with control siRNA or siRNAs against each lncRNA. **p < 0.01, ***p < 0.001, one-way ANOVA. Similarly, significantly reduced colony formation was detected in two separate experiments after these lncRNAs were silenced.

Each novel lncRNA was silenced with three different siRNAs, and the cell proliferation rate was analyzed using the Alamar Blue assay (see Methods). Decreased cell proliferation was detected after the silencing of lncRNAs *TCONS_l2_00001282*, *lnc-GBMT-6,* and *lnc-NBN-1* (Fig. 3A-B and Supplementary Fig. 6A-B). We then investigated whether the silencing of lncRNAs *TCONS_l2_00001282*, *lnc-GBMT-6,* or *lnc-NBN-1* affects colony formation in a clonogenic assay, which revealed decreased colony formation (Fig. 3C-D, Supplementary Fig. 7A). These were the same lncRNAs whose silencing decreased proliferation in the previous assay.

Consistently, lncRNA *TCONS_l2_00001282* had the highest expression in grade 4 IDHmut astrocytomas and GBMs, and its high expression was associated with poorer patient survival in the whole cohort (p < 0.0001, log-rank test) (Fig. 2A and B). The expression of *lnc-NBN-1* was greater in grade 2–3 IDHmut astrocytomas than in other tumor categories (Fig. 2A), and its high expression was associated with better overall survival in the whole cohort (p < 0.0001, log-rank test) (Fig. 2B) but not within any of the diffuse glioma subtypes (Supplementary Fig. 5B–D). Furthermore, *lnc-GBMT-6* showed a tumor type-dependent expression pattern, with the highest expression in oligodendrogliomas and the lowest in GBMs, and consistently, its higher expression was associated with better patient survival in the whole diffuse glioma cohort (p < 0.0001, log-rank test) but not within different tumor entities. To further study the connection of these three lncRNAs to cell cycle, we analyzed how their expression correlates with cell proliferation rate estimated based on mitosis gene set activities. (Supplementary Fig. 8A–C). In IDHmut astrocytomas, *TCONS_l2_00001282* expression moderately positively correlated with proliferation (pearson correlation coefficient 0.34 and 0.33 for S-phase and G2-M phase gene sets, respectively, p < 0.05) and a trend towards correlation was observed for *lnc-GBMT-6* (pearson correlation 0.14 and 0.15 for S-phase and G2-M phase, respectively, p < 0.05). None of the studied lncRNAs was positively associated with cell proliferation in GBM and, for *lnc-GBMT-6*, a trend towards negative correlation was observed (Supplementary Fig. 8B). The results suggest that the studied three lncRNAs are not likely to be mitosis-related genes, at least in GBMs, which would be upregulated during active cell growth. Despite these differences, all three lncRNAs were expressed in most of the tumor samples, and based on our functional experiments, their normal expression was needed for optimal cell growth and colony formation in the GBM cell line SNB19.

To further characterize *TCONS_l2_00001282*, *lnc-GBMT-6*, and *lnc-NBN-1* lncRNAs, we studied their secondary structures using a prediction tool developed by Sato et al.^25^. All the lncRNAs were predicted to harbor known functional structures, such as hairpin loops and internal loops (Supplementary Fig. 9A). These lncRNAs, *TCONS_l2_00001282* (located in the 1p arm), *lnc-GBMT-6* (located in the 5q arm) and *lnc-NBN-1* (located in the 8q arm), are not located near protein-coding genes. As lncRNAs often function through regulating other genes, we studied which genes show correlating expression in diffuse gliomas. When correlations were studied using the strict criteria of Pearson correlation > 0.6 and p < 0.05, we discovered eight genes correlated with *lnc-NBN-1* (Supplementary Table 1). Some of these were lncRNAs, including *RP11, NPIPL1,* and *C1orf191,* which were previously reported to be overexpressed in cancer^26,27^. Additionally, a corresponding protein-coding gene, PRSS55, has been reported to be a possible cancer gene^28^.

## Discussion

We used an in-house developed method to discover putative novel lncRNAs from GBM and grade 4 IDHmut astrocytoma samples in the TCGA GBM cohort and selected 22 of them for additional analysis after manual inspection of gene structures. We showed that these lncRNAs were expressed not only in GBMs but also in IDHmut astrocytomas and oligodendrogliomas. In addition, these lncRNAs were only very lowly expressed or did not show any expression in the normal brain. We also uncovered that the expression of eight lncRNAs was associated with overall patient survival when analyzed within different tumor entities and 14 in the whole diffuse glioma cohort. Furthermore, three of the analyzed lncRNAs (*TCONS_l2_00001282*, *lnc-GBMT-6*, and *lnc-NBN-1*) were functionally relevant, as silencing them led to reduced cell growth and colony formation, whereas three other tested lncRNAs did not have a similar effect.

Several ncRNAs, especially lncRNAs, have been reported to have altered expression in GBMs^16^. Some of them, such as *HOTAIR*, *MALAT1*, and *MEG3,* have even been suggested to act as prognostic factors or therapeutic targets in GBMs^29–31^. Consistently, we detected tumor grade and tumor type-related differences in lncRNA expression and associations with patient survival. Interestingly, different expression patterns and survival associations were observed for different lncRNAs. These results are also linked to each other, as diffuse glioma types have different prognoses: patients with oligodendrogliomas tend to live the longest, patients with IDHmut astrocytomas have a poorer prognosis, and patients with GBM have the shortest life expectancy^24^. Additionally, a higher tumor grade is associated with poorer overall survival^24^. These two known facts were reflected in our results, as 12/14 lncRNAs, for which we observed an association with survival, had significant expression changes when comparing grades within one diffuse glioma type. Additionally, 12/14 lncRNAs presented significant expression changes when different diffuse gliomas of the same grade were compared. It is also possible that the observed expression patterns are partly linked to the neural cell differentiation state that the different tumor types reflect^32–34^. GBMs are considered to be in a lower cell differentiation state than IDH-mutant tumors are, whereas tumor cells in oligodendrogliomas are more likely to represent the cells in the oligodendrocyte differentiation lineage and those in IDHmut astrocytomas in the astrocyte differentiation trajectory^32–34^. We identified four lncRNAs that were associated with longer survival in patients with GBM but several others in other tumor types or when all diffuse gliomas were considered together. Interestingly, all the lncRNAs that were significantly associated with survival within one tumor entity were associated with better patient survival. Our results suggest that these lncRNAs also play roles in other diffuse gliomas, although they were initially discovered in grade 4 tumors. Similar notions have been reported, *e.g.,* for the lncRNA *NEAT1*^35^.

We were able to show that the silencing of *TCONS_l2_00001282*, *lnc-GBMT-6*, and *lnc-NBN-1* led to reduced cell proliferation and colony formation. However, high expression of *lnc-GBMT-6* and *lnc-NBN-1* was associated with better patient survival in the full TCGA diffuse glioma cohort. This discrepancy between survival data and cell line data is understandable, as *lnc-GBMT-6* has the highest expression in oligodendrogliomas and the lowest expression in GBM, which are associated with the longest and shortest survival, respectively. Furthermore, most of the cases with low *lnc-GBMT-6* expression (below the median) were GBMs and grade 4 IDHmut astrocytomas, which are associated with the poorest prognosis among diffuse gliomas. Almost all GBM samples in the TCGA cohort show expression of *lnc-GBMT-6* and *lnc-NBN-1.* These lncRNAs may be needed for GBM cell growth and colony formation, as our cell line experiments revealed, but their upregulation is not associated with a more aggressive tumor phenotype. This is also supported by the higher expression of these lncRNAs in tumor samples than in the normal brain.

Possible future aspects could include overexpression of selected lncRNAs in the glioblastoma cell models. With overexpression experiments, it would be possible to see whether higher levels of *lnc-GBMT-6* or *lnc-NBN-1* could also inhibit growth, which would explain the observed survival effect in TCGA data, increase proliferation, or show no effect. At least correlation analysis in respect to cell proliferation rate did not provide evidence on the additive effect of these lncRNAs in GBM, suggesting that their overexpression would not enhance proliferation. It is good to keep in mind that the effect of siRNA-mediated silencing can be stronger and not solely depend on lower expression levels, as siRNAs can also affect the function of lncRNAs by disturbing their structure via binding^36^. In summary, *lnc-GBMT-6* or *lnc-NBN-1* appear to be needed for optimal cell growth but they do not necessarily have an additive effect above a certain expression level.

Gene *TCONS_l2_00001282* has an exon structure and initiator motif in the promoter region. We also observed several hairpin loops, which are known to play roles in regulation, in the secondary structure of *TCONS_l2_00001282*^37^. Gene *lnc-GBMT-6* also has an exon structure but harbors a TATA-box motif in the TSS region. It was also expressed in all four GBM cell lines studied and has several hairpin loops in its secondary structure. Finally, *lnc-NBN-1* has an exon structure and a secondary structure with hairpin loops and its expression was correlated with several other genes that are connected to cancer. Together, these characteristics suggest that these lncRNAs play active roles in malignant cells.

In this study, we identified lncRNAs expressed in GBMs, some of which presented altered expression in diffuse gliomas, and some were associated with patient survival in different tumor entities or when all diffuse gliomas were considered together. We also showed that three of the lncRNAs had functional effects when silenced in the GBM cell lines. Interestingly, most of the lncRNAs we identified were novel, and others have been reported previously. Further research is needed to more precisely define the mechanisms of function of the identified lncRNAs and to determine whether other lncRNAs have relevant functions in these tumor types.

## Materials and methods

### Discovery of ncRNAs

Altogether 169 raw RNA-sequence files from GBMs and grade 4 IDH-mutant astrocytomas (diagnosed previously as GBMs) were acquired from TCGA and aligned with TopHat (using default parameters) against a reference genome (version hg19). SAMtools was used to create sorted and indexed, binary files (BAM files). The RPM (reads per million mapped reads)- normalized read coverage values were then calculated, using a 500 bp window and a 250 bp sliding step. For each window, the highest and lowest 20% of the expression is compared to the median and if the absolute difference is > 1 and the fold change |log2(fc)|> 1, a window is chosen. Known genes are filtered with information from Ensembl, Refseq, UCSC, and Gencode. Overlapping significant windows are merged. Transcripts are scored based on promoter, 3’UTR, ORFs, and exon–intron structure. The transcripts were inspected in IGV^38^. The promoter score is calculated using GC enrichment around the TSS:

$$S_{GC} = \left\{ {\begin{array}{*{20}l} {0,} \hfill & {\% (G + C) < 0.4} \hfill \\ {\frac{\% (G + C) - 0.4}{{0.15}},} \hfill & {0.4 \le \% (G + C) \le 0.55} \hfill \\ {1,} \hfill & {\% (G + C) > 0.55} \hfill \\ \end{array} } \right.$$

Chargaff’s second rule violation:

$$S_{Charg} = \left\{ {\begin{array}{*{20}l} {0,} \hfill & {\% (G + T) < 0.5} \hfill \\ {\frac{\% (G + T) - 0.5}{{0.1}},} \hfill & {0.5 \le \% (G + T) \le 0.6} \hfill \\ {1,} \hfill & {\% (G + T) > 0.6} \hfill \\ \end{array} } \right.$$

Motif existence (TATA, DPE, or Initiator):

$$S_{motif} = \left\{ {\begin{array}{*{20}l} {1,} \hfill & {{\text{perfect}}\;{\text{match}}\;{\text{found}}\;{\text{for}}\;{\text{any}}\;{\text{of}}\;{\text{the}}\;{\text{motifs}}} \hfill \\ {0,} \hfill & {\text{otherwise,}} \hfill \\ \end{array} } \right.$$

The total promoter score is then calculated:

$$S_{prom} = \frac{{S_{motif} + S_{GC} + S_{Ch\arg } }}{3}$$

The 3’UTR score is calculated by checking if there is a poly-A tail followed by a T-rich region. First, the poly-A motif is searched and then, the score is calculated: (near-perfect match is a single nucleotide mismatch)

$$S_{polyA} = \left\{ {\begin{array}{*{20}l} {1,} \hfill & {{\text{perfect}}\;{\text{match}}\;{\text{found}}} \hfill \\ {0.5,} \hfill & {{\text{near}}\;{\text{perfect}}\;{\text{match}}\;{\text{found}}} \hfill \\ {0,} \hfill & {\text{otherwise,}} \hfill \\ \end{array} } \right.$$

The T-rich region is then calculated up to 80 bp downstream from where the poly-A site was found:

$$S_{T - rich} = \left\{ {\begin{array}{*{20}l} {0,} \hfill & {\% T < 0.3} \hfill \\ {\sqrt {\frac{\% T - 0.3}{{0.1}},} } \hfill & {0.3 \le \% T \le 0.4} \hfill \\ {1,} \hfill & {\% T > 0.4.} \hfill \\ \end{array} } \right.$$

The total 3’UTR score is then calculated:

$$S_{{3^{\prime } UTR}} = \frac{{S_{polyA} + S_{T - rich} }}{2}$$

The ORF score is calculated where n_ORF_ is the number of exons in the longest ORF, n_ex_ is the total number of exons and p is 0.5 if the last exon is included in the ORF, otherwise, it is 0.

$$S_{ORF} = \left( {\sqrt {\frac{{n_{ORF} }}{{n_{ex} }}} + p} \right)/1.5,$$

The exon score is calculated as follows; n_valid_ is the number of exons with valid splice junctions and n_ex_ is the total number of exons:

$$S_{ex} = \left\{ {\begin{array}{*{20}l} {0,} \hfill & {n_{ex} = 1} \hfill \\ {\frac{{n_{valid} }}{{n_{ex} }},} \hfill & {n_{ex} > 1,} \hfill \\ \end{array} } \right.$$

Finally, the scores are summed, and the candidate transcripts are ranked based on that score.

### TCGA RNA-seq expression and survival analysis

Raw sequencing reads from RNA-seq experiments of samples in the GBM and LGG cohorts generated by TCGA were downloaded from the National Cancer Institute (NCI) Genomic Data Commons (GDC) legacy database. The RNAseq reads were aligned to the UCSC human genome hg19 using TopHat with default parameters, and read counts were computed for the lncRNAs based on their genomic coordinates. Only cases with information about the IDH mutation and 1p19q status were included in the analysis. According to the WHO 2021 classification of CNS tumors, IDH wild-type grade 2 and 3 tumors with chromosome 7 gain, chromosome 10 loss, TERT promoter mutation, or EGFR amplification were considered GBMs ^3^, leaving a total of 559 TCGA cases with expression data. Tumors were then categorized into tumor entities based on *IDH* mutation status and 1p19q codeletion status and IDH-mutant tumors further based on tumor grade. By definition, all GBMs are grade 4. All the IDH-mutant astrocytomas with full CDKN2A deletion were considered grade 4. Significant differences in log2-transformed RNA expression levels between glioma subtypes with the same tumor grade or between tumor grades within one tumor entity were calculated in 559 TCGA cases (141 IDH-mutant oligodendrogliomas, 213 IDH-mutant astrocytomas, and 205 GBMs) using the Wilcoxon rank-sum test. The associations between survival and high vs. low log2 expression status (median expression as a cutoff) were tested using a log-rank test including only primary tumors (540 TCGA cases) and visualized with Kaplan–Meier plots using the R packages survival and survminer. The relationship of lncRNAs with cell cycle genes overexpressed in S- and G2/M-phase were analyzed as in Rautjoki et al.^39^. Pearson and Spearman correlations of lncRNA expression with gene set activity Z-scores (calculated as the average of Z-scores of individual genes)^40^ were calculated within TCGA tumor subtypes. Five tumors from each diffuse glioma subtype were used for visualizing the expression patterns and gene structures with IGV.

### Cell line experiments

The glioblastoma cell lines U-118, T98G, and LN229 were obtained from the American Type Culture Collection. SNB19 cells were obtained from the Wei Zhang laboratory in University of Texas M.D. Anderson Cancer Center, Houston, TX, United States. All the cell lines were maintained in Dulbecco’s modified Eagle’s medium (Gibco REF:41966-029) supplemented with 10% fetal bovine serum (Sigma REF:F7524). The cells were cultured at 37 °C with 5% CO2.

The SNB19 cell line was used for functional experiments. The cells were grown to 75% confluence on plates and transfected with Lipofectamine RNAiMAX Reagent. Transfection was performed according to the Lipofectamine protocol at a 20 µM concentration for each of the three target siRNAs which were pooled together (Supplementary Table 1).

For the cell proliferation experiments, 20 000 cells were seeded the day after transfection into a 24-well plate. Each condition was performed in six replicates. Each day, cell viability was measured using AlamarBlue (REF DAL1100) with the AlamarBlue protocol and AlamarBlue reagent, with pure cell growth medium used as a control. The plates were read using a Perkin Elmer UV/VIS Envision 2104 plate reader with 570 nm excitation and 585 nm emission wavelengths.

For clonogenic assay, 400 cells were seeded on the day after transfection into a 6-well plate. Each condition was performed in triplicate. The medium was changed twice, and after two weeks, the medium was removed, and the cells were washed 3 × with PBS, fixed with 10% neutral buffered formalin solution, and stained with crystal violet (0.5% w/v), after which the colonies were counted manually.

### PCR and qPCR

Total RNA was isolated using a mirVana isolation kit (Invitrogen). The RNA was converted to cDNA with random primers and SuperScript III Reverse Transcriptase (Life Technologies). PCR was performed with Q5 DNA polymerase. The primers used for each of the 22 transcripts are listed in Supplementary Table 1. qPCR was performed with SYBR Green and a Bio-Rad CFX96 Touch Real-Time PCR qPCR system.

### Normal brain analysis

The Genotype-Tissue Expression (GTEx) Project data was obtained from GTEx Portal for 21 participants that had RNA-seq data from 7 sample locations (anterior cingulate cortex, cerebellar hemisphere, cerebellum, cortex, frontal cortex, hippocampus, spinal cord) where glioblastoma can develop. These 147 GTEx samples were used for comparison between tumor (TCGA-GBM and TCGA-LGG) and normal tissue expression of lncRNAs. lncRNA coordinates were lifted over to hg38 genome and bedtools multicov was used for counting the lncRNA alignments, which were RPM normalized.

## Supplementary Information

Supplementary Information 1.

Supplementary Information 2.

## Data Availability

TCGA data availability: TCGA expression data is available in the NIH database of Genotypes and Phenotypes dbGaP under the accession number phs000178.v11.p8: The Cancer Genome Atlas (TCGA). The GTEx datasets used for the analyses described in this manuscript were obtained from dbGaP at http://www.ncbi.nlm.nih.gov/gap through dbGaP accession number phs000424.v10.p2 : Common Fund (CF) Genotype-Tissue Expression Project (GTEx). All other data supporting the findings of this study are available within the paper and its Supplementary Information.
