# Supplementary material for: Tumor-associated long non-coding RNAs show variable expression across diffuse gliomas and effect on cell growth upon silencing in glioblastoma
Source: Sci Rep. 2025 May 9;15:16220. doi: 10.1038/s41598-025-99984-9 (PMC12064817; doi:10.1038/s41598-025-99984-9)
Supplement: Supplementary file 1 — Supplementary Information 1. [file 41598_2025_99984_MOESM1_ESM.pdf]

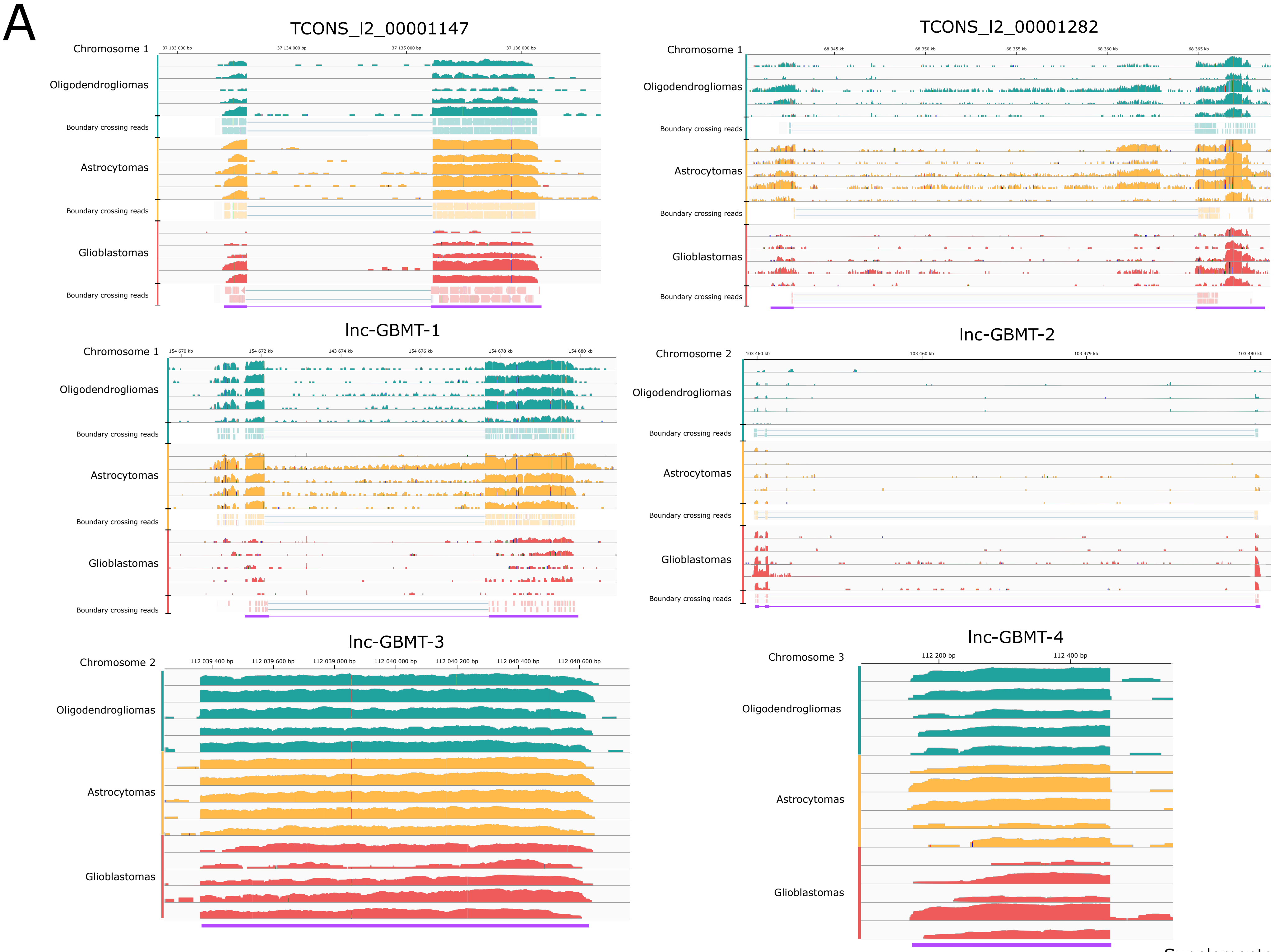

Supplementary Figure 1

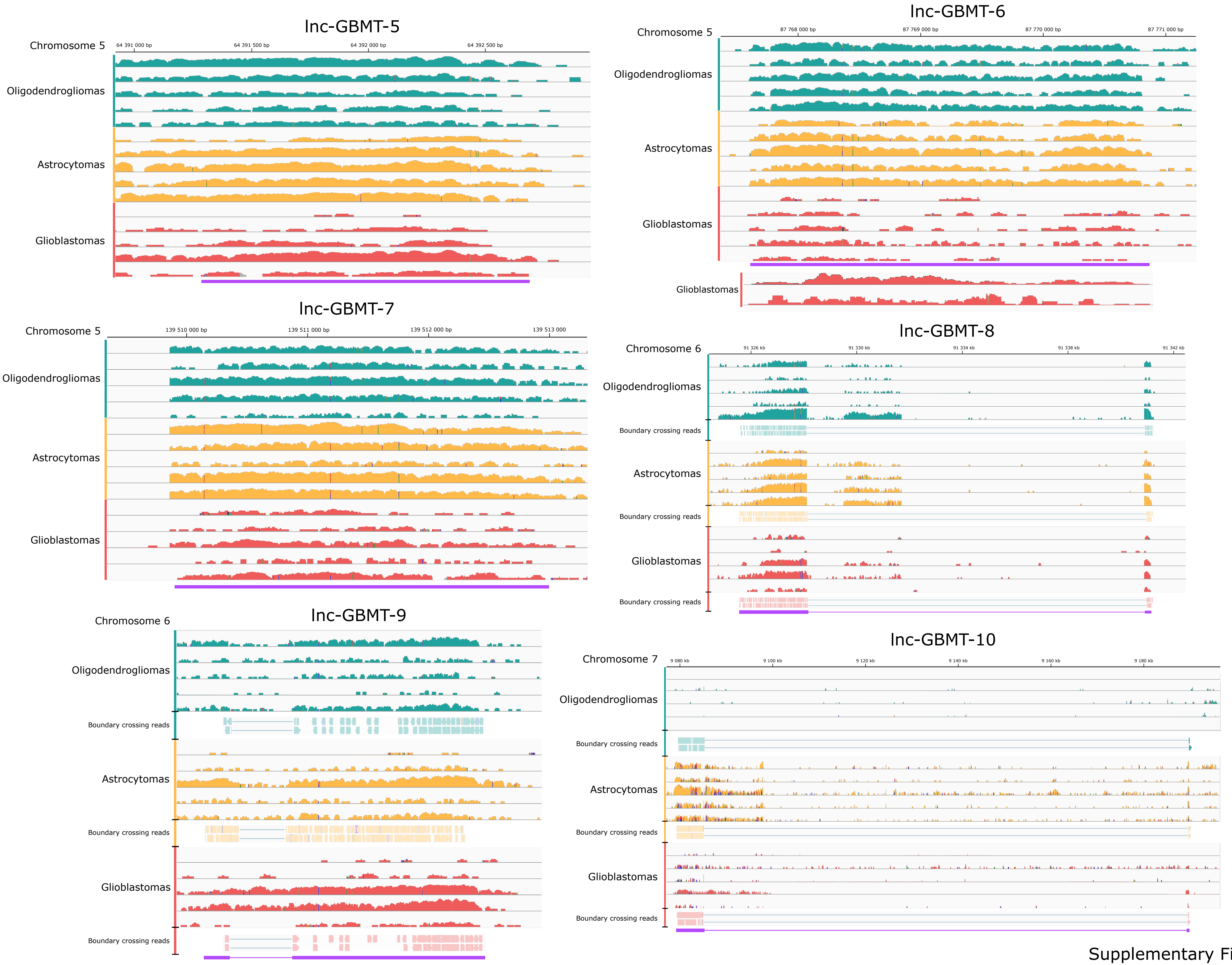

Supplementary Figure 1

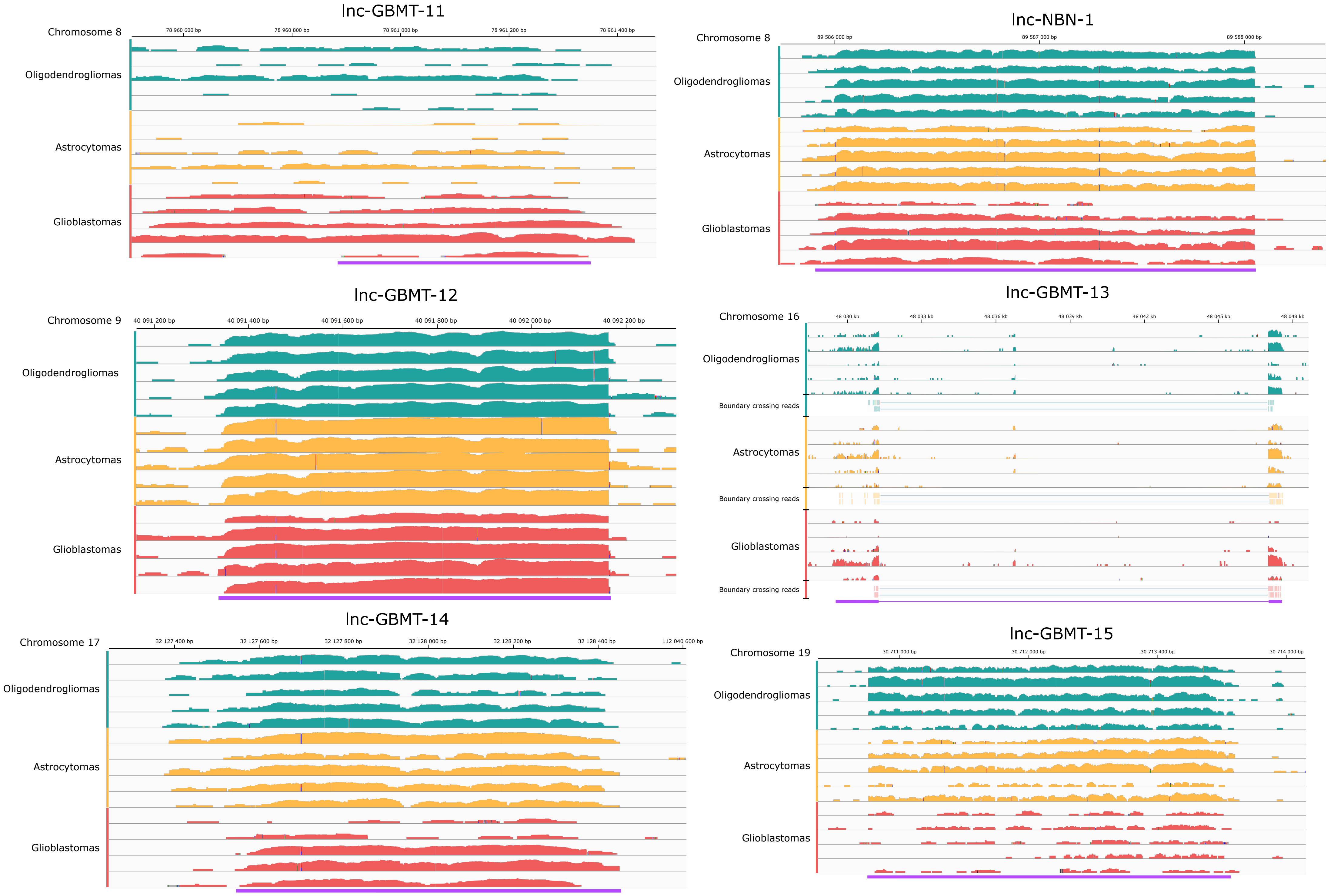

Supplementary Figure 1

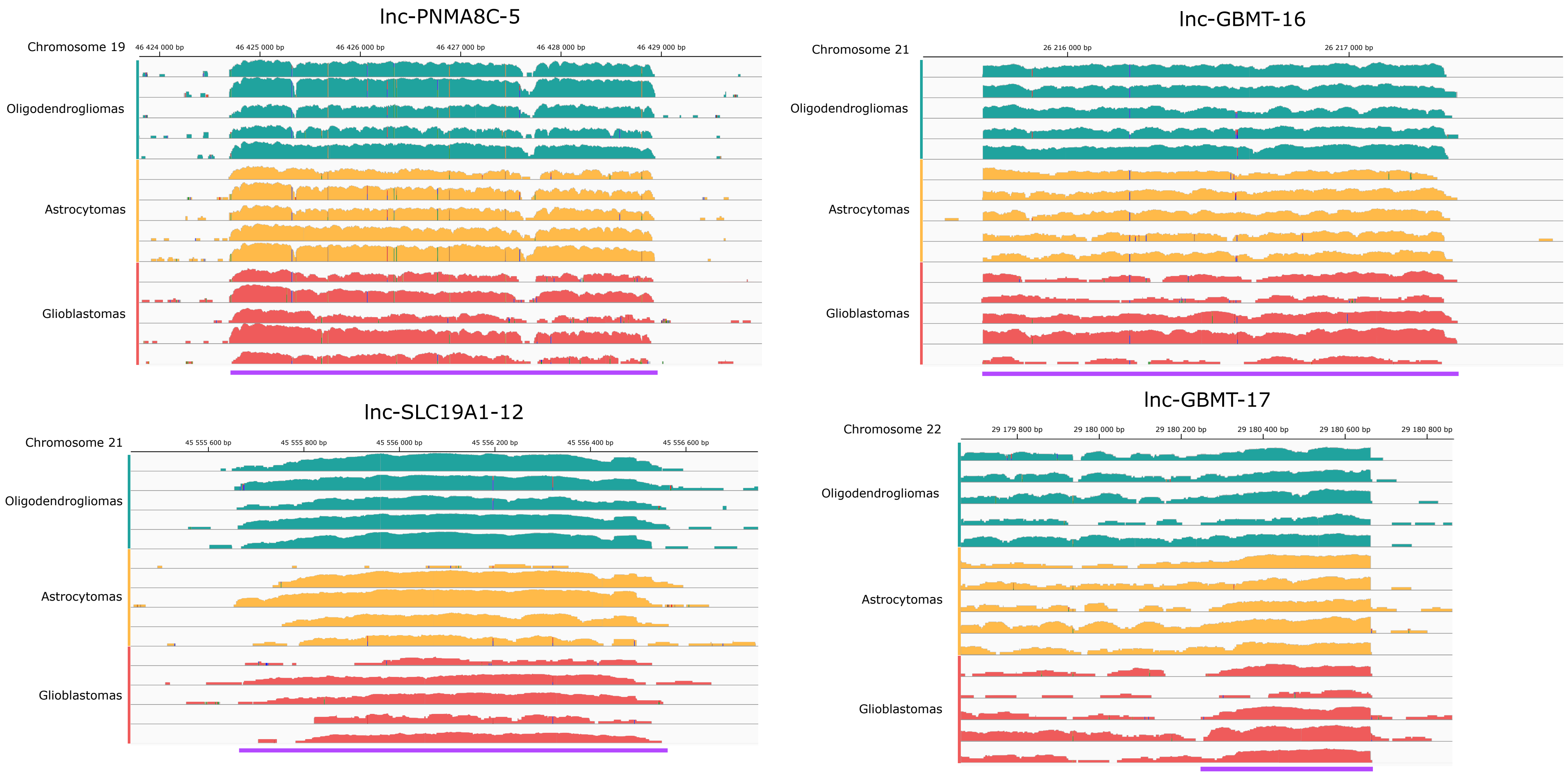

Supplementary Figure 1: a) Representative figures of five samples from each diffuse gliomas were selected and Interactive Genomics Viewer (IGV) snapshot was taken for the location of each of the 22 lncRNAs. Coordinates are in genome build hg38. Scale of each track is set to 0 to 150 on logarithmic scale. For those lncRNAs where there are several exons, boundary crossing reads are presented for each diffuse glioma. Purple bar represents the lncRNA location. For lnc-GBMT-6, two extra GBM samples with higher gene expression from TCGA were added to provide further evidence on its gene structure in GBM. Custom scale was also adjusted to these two tracks.

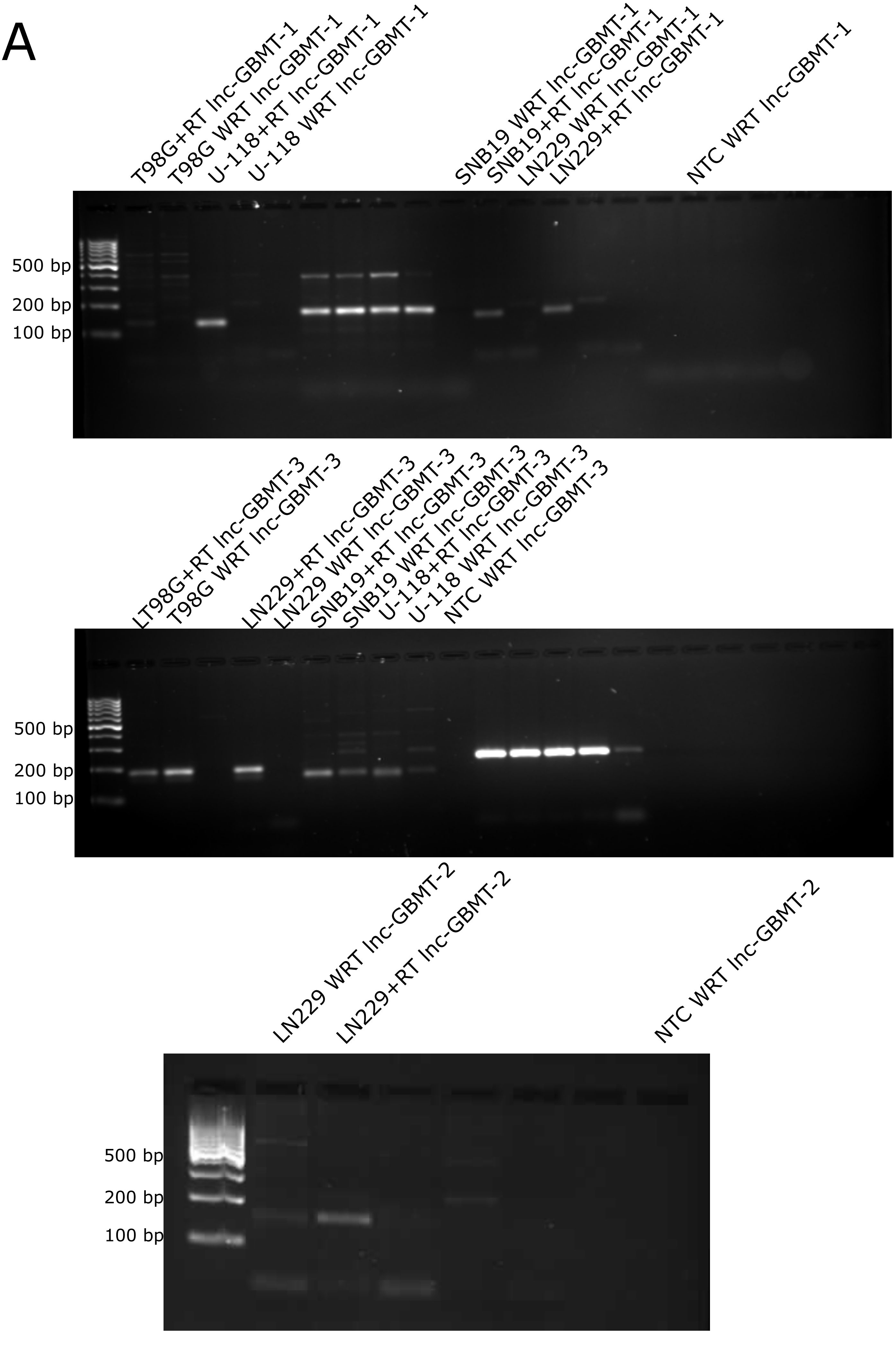
A
T98G+RT lnc-GBMT-1
T98G WRT lnc-GBMT-1
U-118+RT lnc-GBMT-1
U-118 WRT lnc-GBMT-1
SNB19 WRT lnc-GBMT-1
SNB19+RT lnc-GBMT-1
LN229 WRT lnc-GBMT-1
LN229+RT lnc-GBMT-1
NTC WRT lnc-GBMT-1
500 bp
200 bp
100 bp
LT98G+RT lnc-GBMT-3
T98G WRT lnc-GBMT-3
LN229+RT lnc-GBMT-3
LN229 WRT lnc-GBMT-3
SNB19+RT lnc-GBMT-3
SNB19 WRT lnc-GBMT-3
U-118+RT lnc-GBMT-3
U-118 WRT lnc-GBMT-3
NTC WRT lnc-GBMT-3
500 bp
200 bp
100 bp
LN229 WRT lnc-GBMT-2
LN229+RT lnc-GBMT-2
NTC WRT lnc-GBMT-2
500 bp
200 bp
100 bp

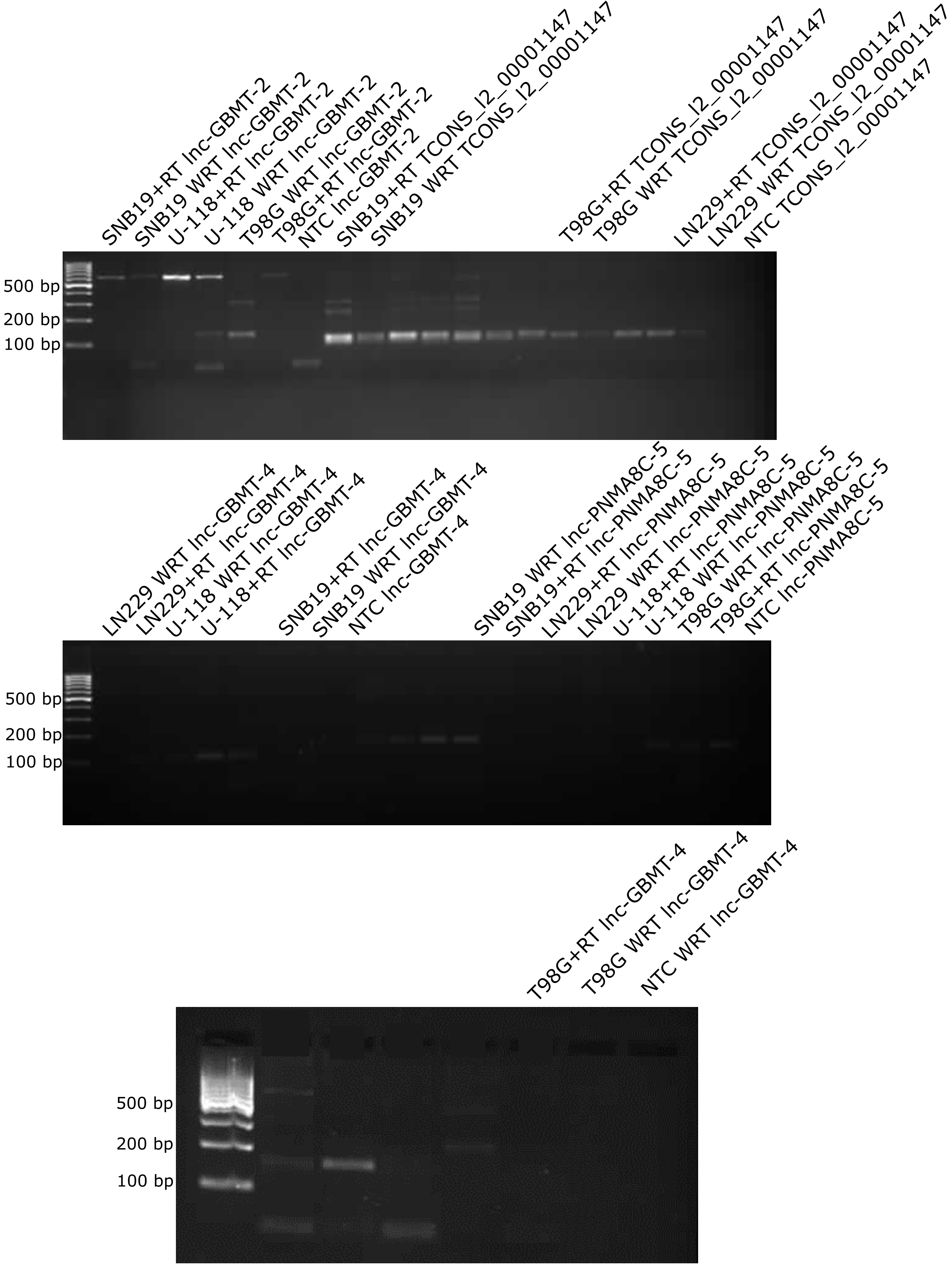
SNB19+RT lnc-GBMT-2
SNB19 WRT lnc-GBMT-2
U-118+RT lnc-GBMT-2
U-118 WRT lnc-GBMT-2
T98G WRT lnc-GBMT-2
T98G+RT lnc-GBMT-2
NTC lnc-GBMT-2
SNB19+RT TCONS_l2_00001147
SNB19 WRT TCONS_l2_00001147
T98G+RT TCONS_l2_00001147
T98G WRT TCONS_l2_00001147
LN229+RT TCONS_l2_00001147
LN229 WRT TCONS_l2_00001147
NTC TCONS_l2_00001147
500 bp
200 bp
100 bp
LN229 WRT lnc-GBMT-4
LN229+RT lnc-GBMT-4
U-118 WRT lnc-GBMT-4
U-118+RT lnc-GBMT-4
SNB19+RT lnc-GBMT-4
SNB19 WRT lnc-GBMT-4
NTC lnc-GBMT-4
SNB19 WRT lnc-PNMA8C-5
SNB19+RT lnc-PNMA8C-5
LN229+RT lnc-PNMA8C-5
LN229 WRT lnc-PNMA8C-5
U-118+RT lnc-PNMA8C-5
U-118 WRT lnc-PNMA8C-5
T98G WRT lnc-PNMA8C-5
T98G+RT lnc-PNMA8C-5
NTC lnc-PNMA8C-5
500 bp
200 bp
100 bp
T98G+RT lnc-GBMT-4
T98G WRT lnc-GBMT-4
NTC WRT lnc-GBMT-4
500 bp
200 bp
100 bp

SNB19+RT lnc-GBMT-5
SNB19 WRT lnc-GBMT-5
LN229+RT lnc-GBMT-5
LN229 WRT lnc-GBMT-5
T98G+RT lnc-GBMT-5
T98G WRT lnc-GBMT-5
NTC lnc-GBMT-5

500 bp
200 bp
100 bp

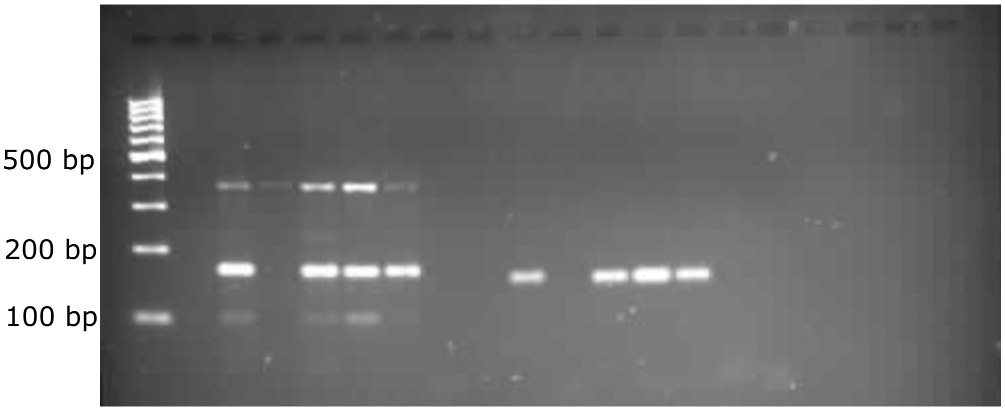

LN229 WRT lnc-GBMT-16
LN229+RT lnc-GBMT-16
U-118 WRT lnc-GBMT-16
U-118+RT lnc-GBMT-16
T98G+RT lnc-GBMT-16
T98G WRT lnc-GBMT-16
SNB19+RT lnc-GBMT-16
SNB19 WRT lnc-GBMT-16
NTC lnc-GBMT-16
NTC lnc-GBMT-6
LN229+RT lnc-GBMT-6
LN229 WRT lnc-GBMT-6
U-118+RT lnc-GBMT-6
U-118 WRT lnc-GBMT-6
SNB19 WRT lnc-GBMT-6
SNB19+RT lnc-GBMT-6

500 bp
200 bp
100 bp

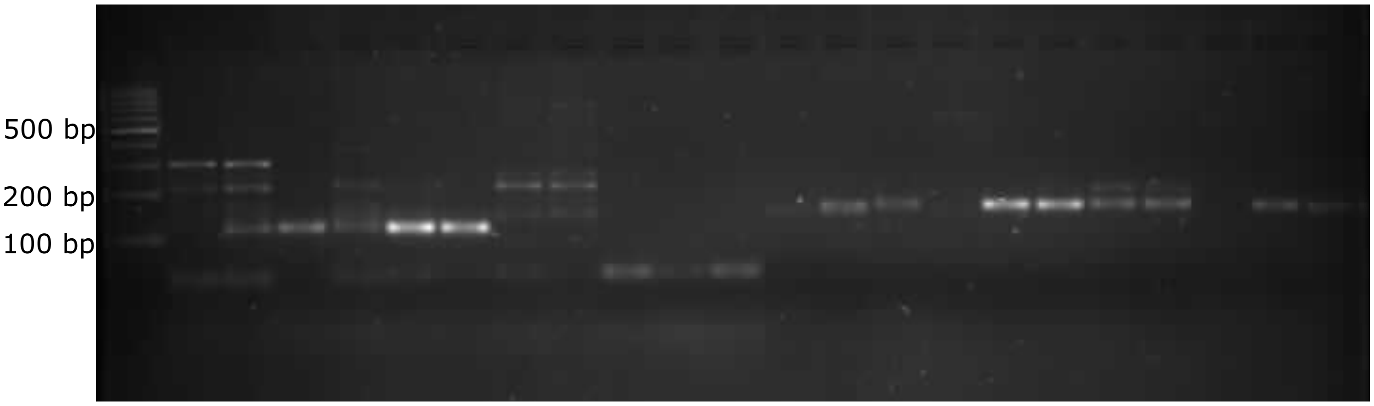

LN229 WRT lnc-GBMT-7
LN229+RT lnc-GBMT-7
SNB19 WRT lnc-GBMT-7
SNB19+RT lnc-GBMT-7
U-118 WRT lnc-GBMT-7
U-118+RT lnc-GBMT-7
NTC WRT lnc-GBMT-7

500 bp
200 bp
100 bp

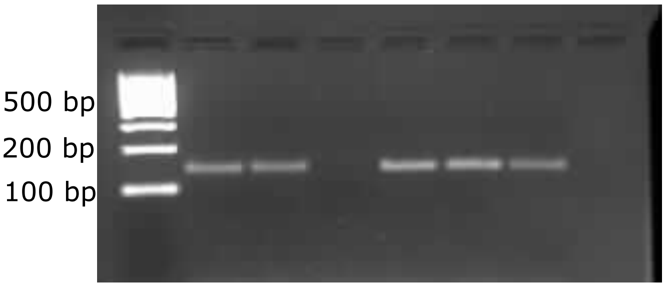

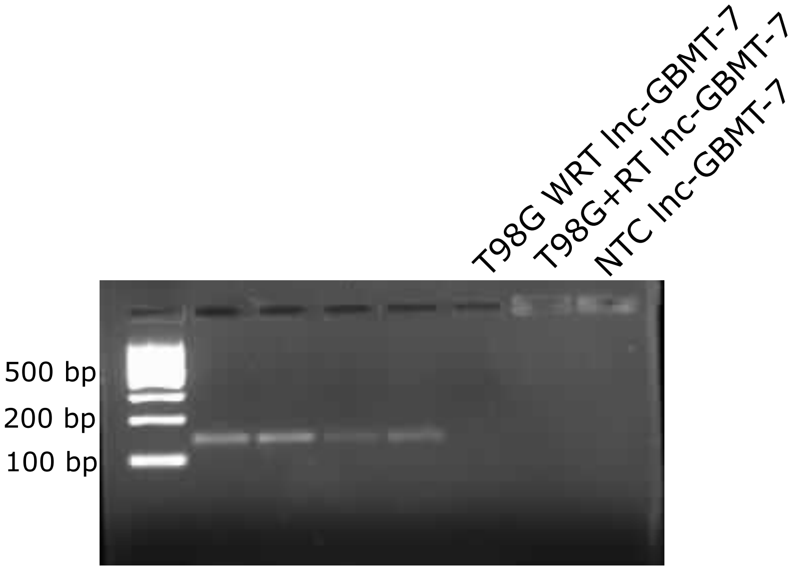
T98G WRT lnc-GBMT-7
T98G+RT lnc-GBMT-7
NTC lnc-GBMT-7
500 bp
200 bp
100 bp

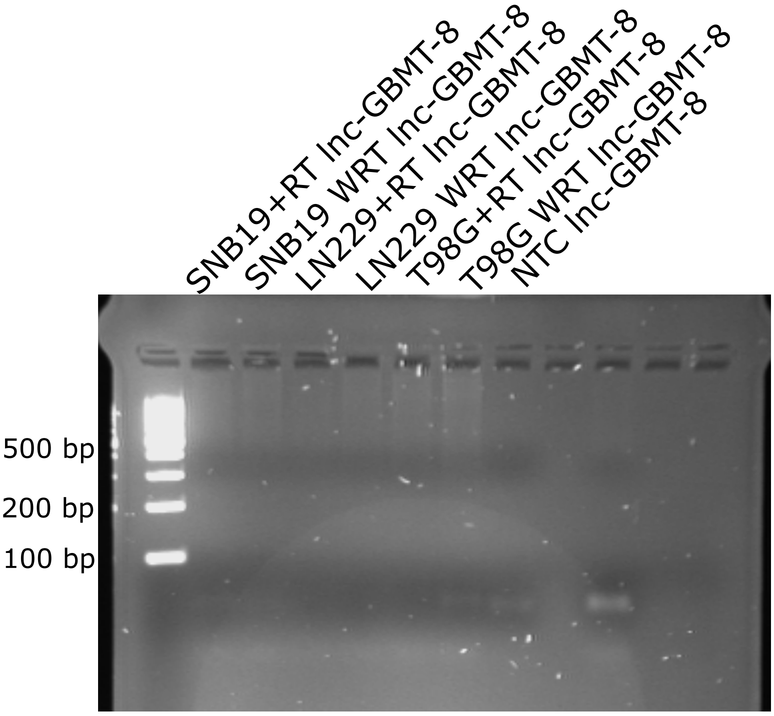
SNB19+RT lnc-GBMT-8
SNB19 WRT lnc-GBMT-8
LN229+RT lnc-GBMT-8
LN229 WRT lnc-GBMT-8
T98G+RT lnc-GBMT-8
T98G WRT lnc-GBMT-8
NTC lnc-GBMT-8
500 bp
200 bp
100 bp

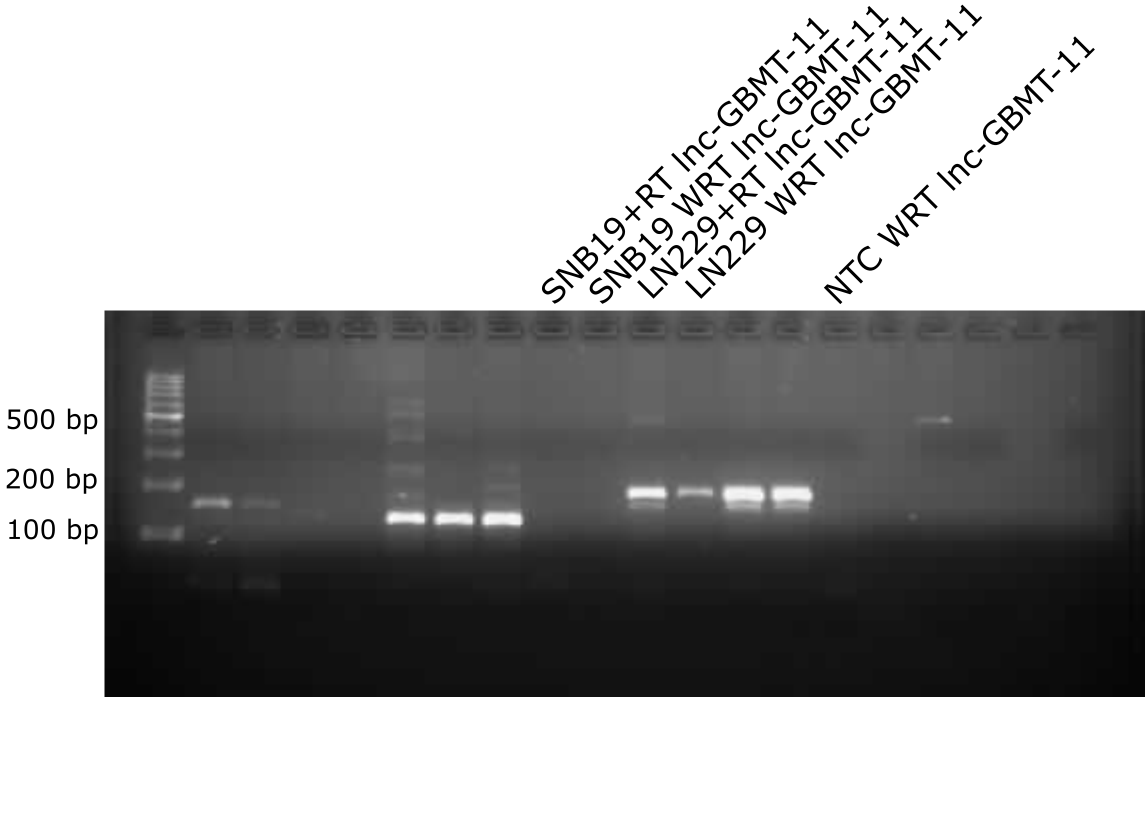
SNB19+RT lnc-GBMT-11
SNB19 WRT lnc-GBMT-11
LN229+RT lnc-GBMT-11
LN229 WRT lnc-GBMT-11
NTC WRT lnc-GBMT-11
500 bp
200 bp
100 bp

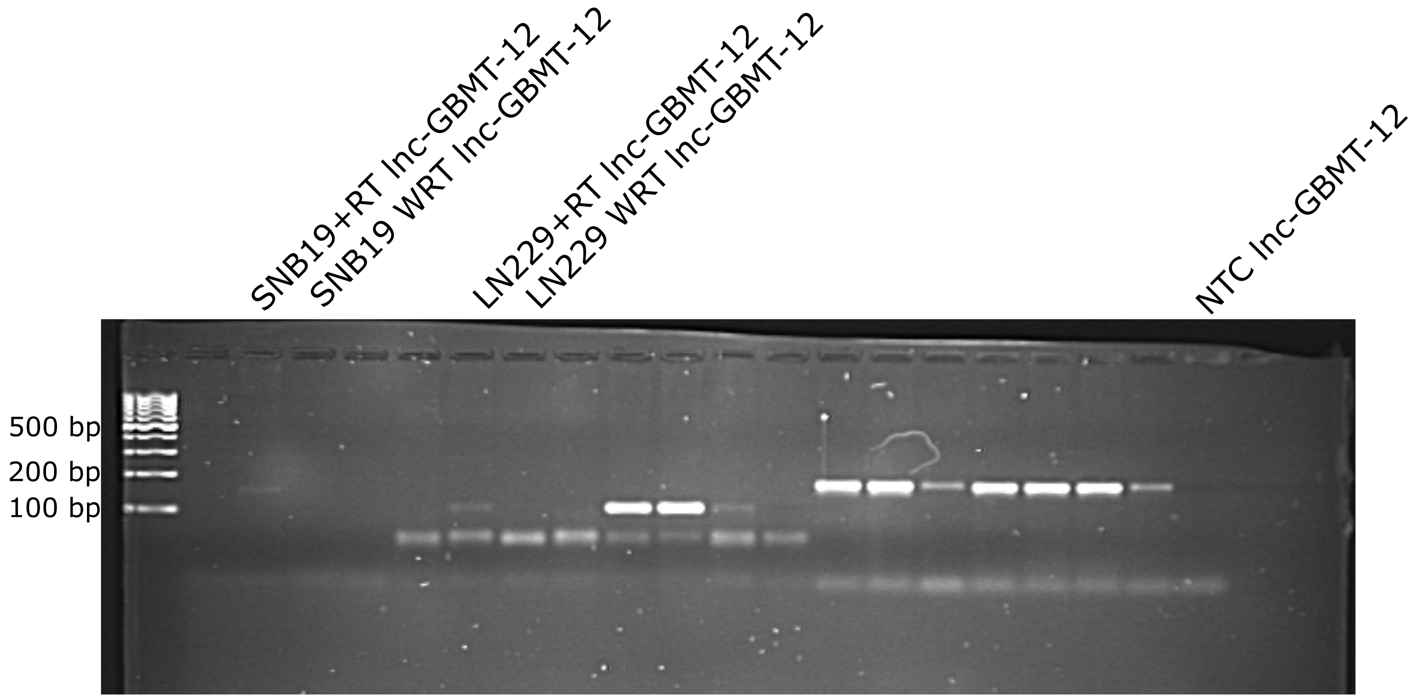
SNB19+RT lnc-GBMT-12
SNB19 WRT lnc-GBMT-12
LN229+RT lnc-GBMT-12
LN229 WRT lnc-GBMT-12
NTC lnc-GBMT-12
500 bp
200 bp
100 bp

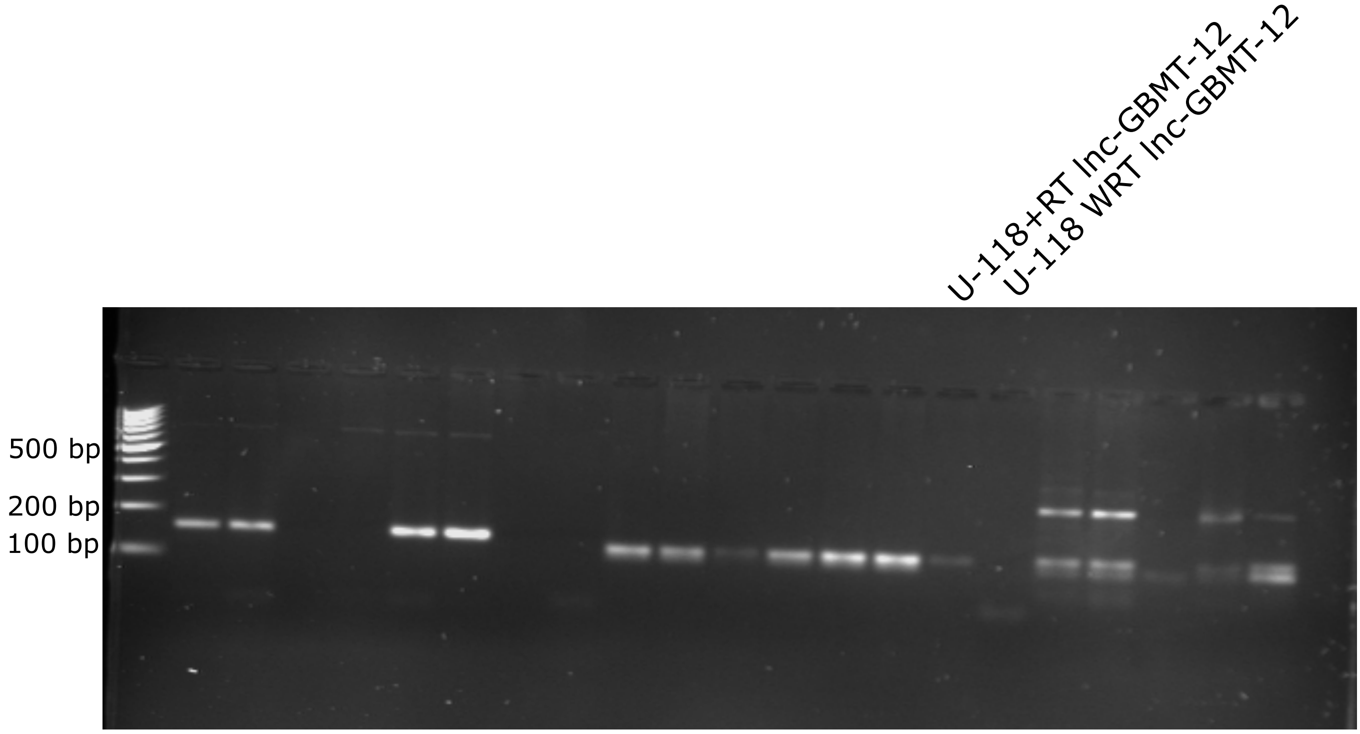
U-118+RT lnc-GBMT-12
U-118 WRT lnc-GBMT-12
500 bp
200 bp
100 bp

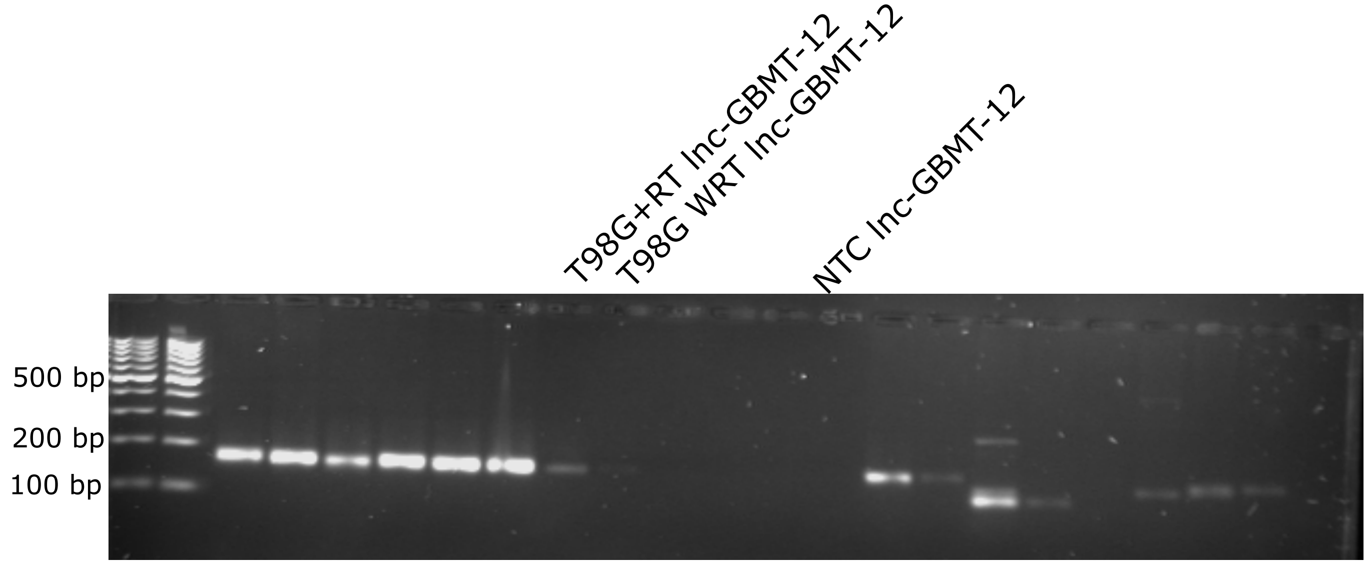
T98G+RT lnc-GBMT-12
T98G WRT lnc-GBMT-12
NTC lnc-GBMT-12
500 bp
200 bp
100 bp

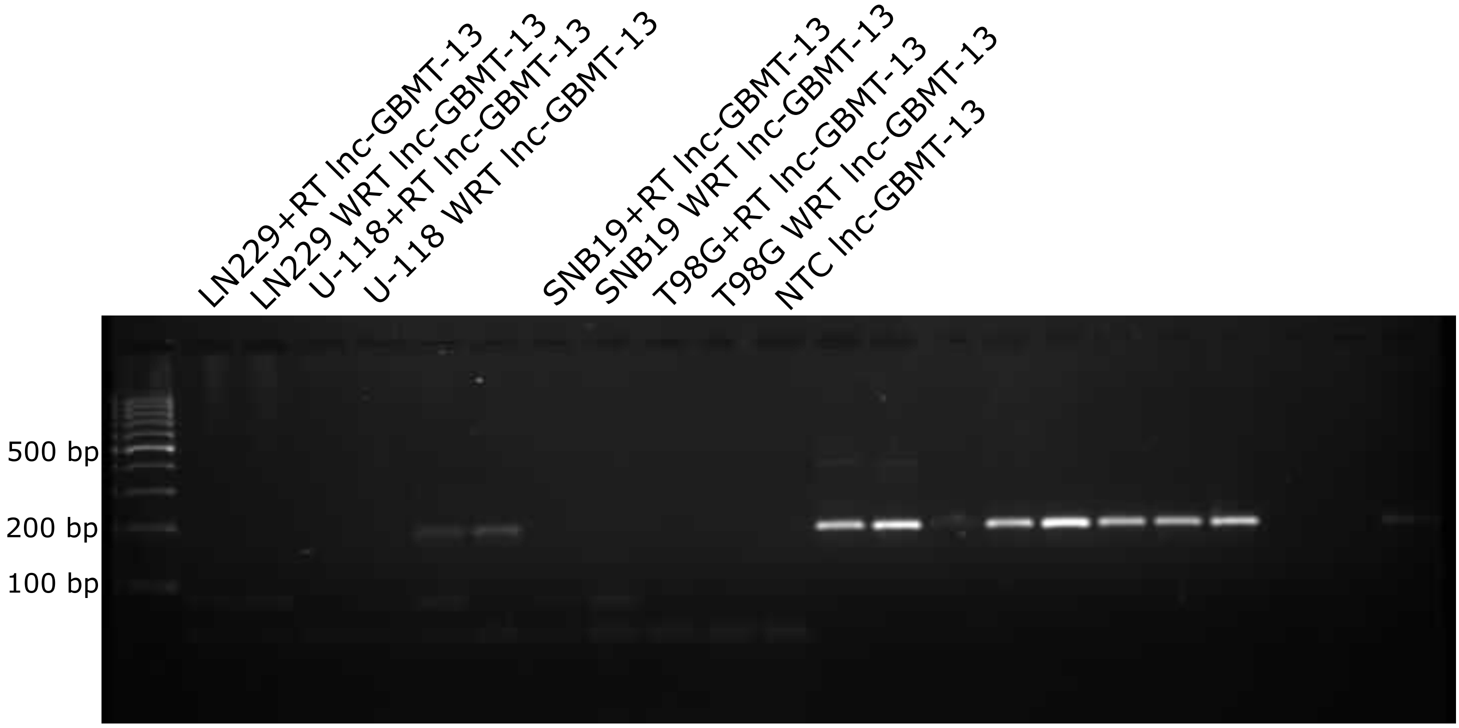
LN229+RT lnc-GBMT-13
LN229 WRT lnc-GBMT-13
U-118+RT lnc-GBMT-13
U-118 WRT lnc-GBMT-13
SNB19+RT lnc-GBMT-13
SNB19 WRT lnc-GBMT-13
T98G+RT lnc-GBMT-13
T98G WRT lnc-GBMT-13
NTC lnc-GBMT-13
500 bp
200 bp
100 bp

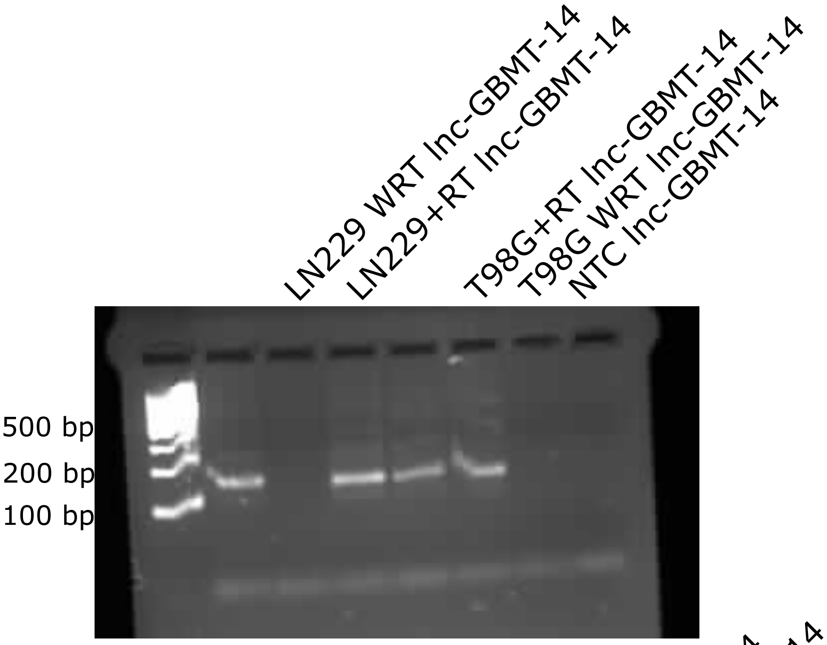
LN229 WRT lnc-GBMT-14
LN229+RT lnc-GBMT-14
T98G+RT lnc-GBMT-14
T98G WRT lnc-GBMT-14
NTC lnc-GBMT-14
500 bp
200 bp
100 bp

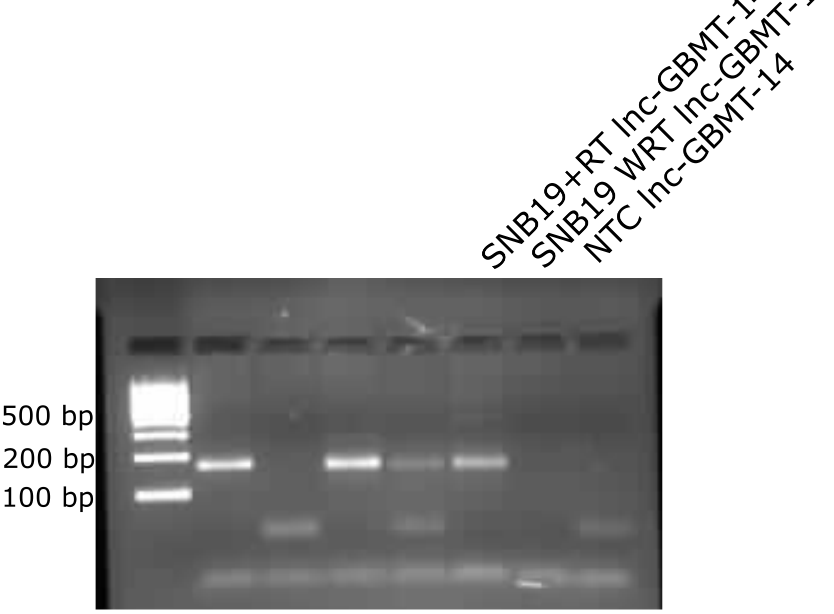
SNB19+RT lnc-GBMT-14
SNB19 WRT lnc-GBMT-14
NTC lnc-GBMT-14
500 bp
200 bp
100 bp

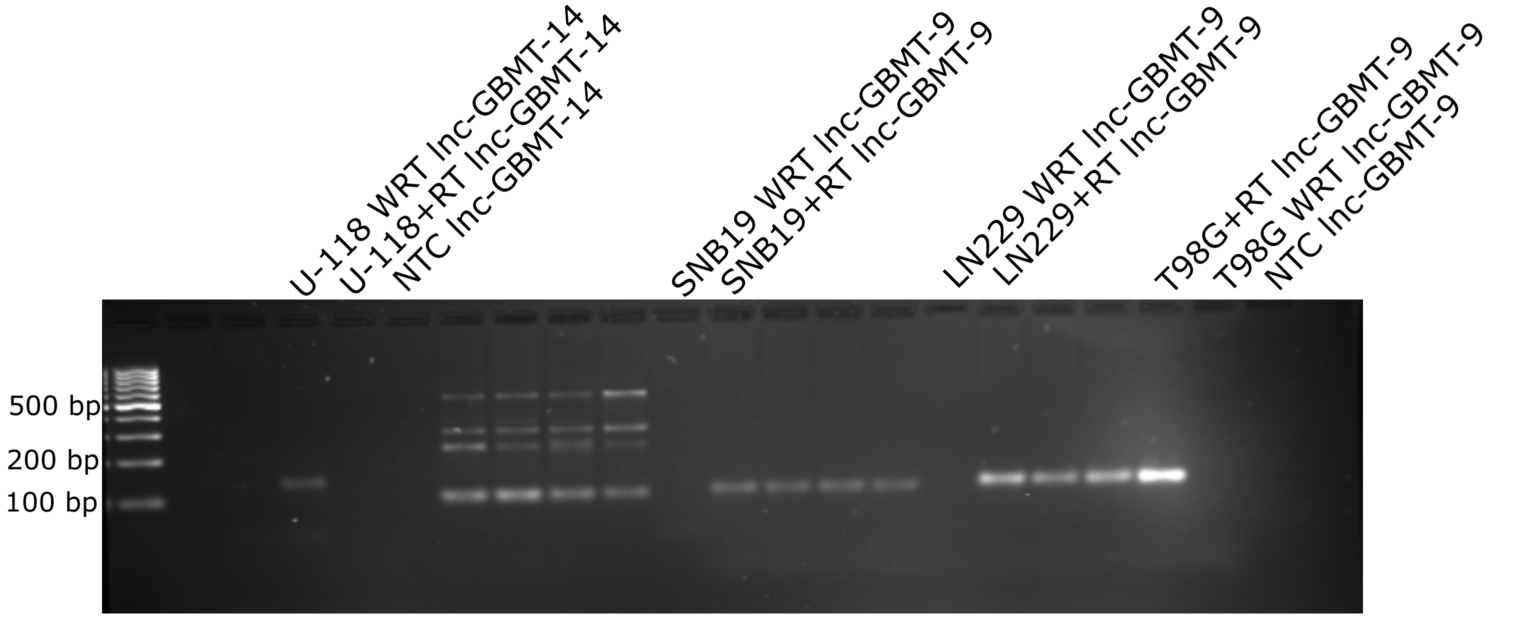
U-118 WRT lnc-GBMT-14
U-118+RT lnc-GBMT-14
NTC lnc-GBMT-14
SNB19 WRT lnc-GBMT-9
SNB19+RT lnc-GBMT-9
LN229 WRT lnc-GBMT-9
LN229+RT lnc-GBMT-9
T98G+RT lnc-GBMT-9
T98G WRT lnc-GBMT-9
NTC lnc-GBMT-9
500 bp
200 bp
100 bp

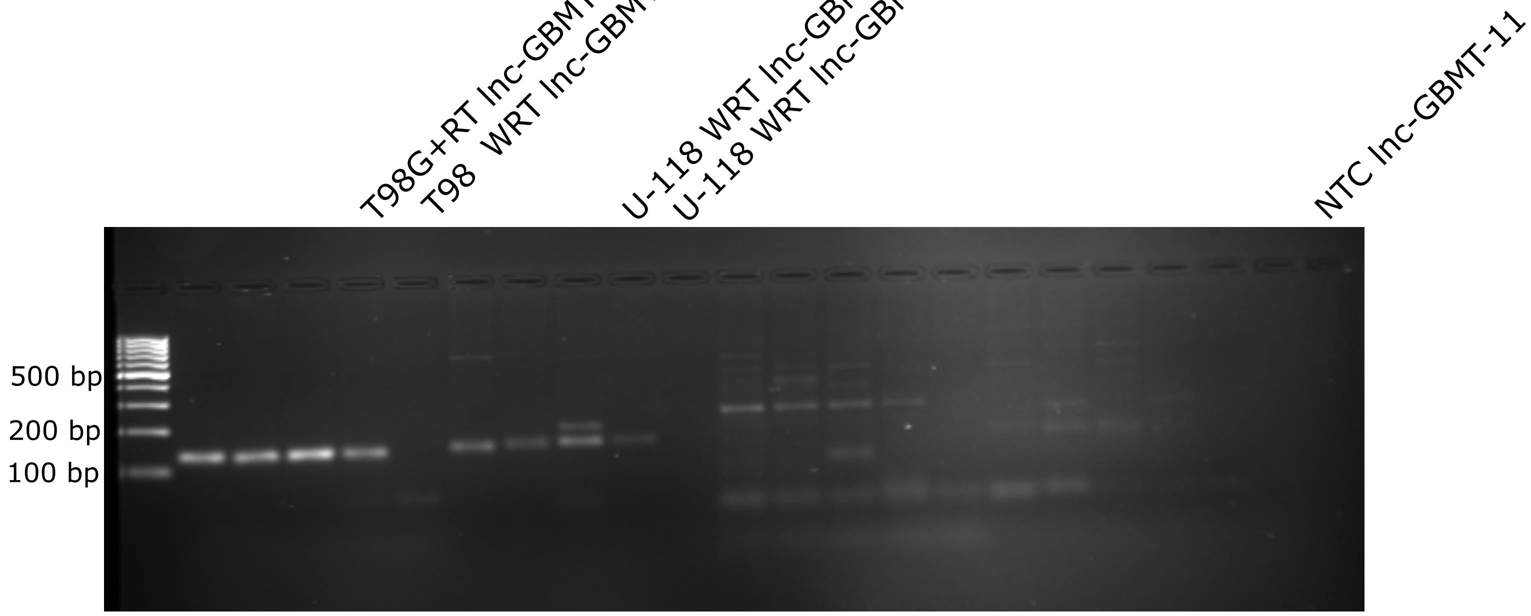
T98G+RT lnc-GBMT-11
T98 WRT lnc-GBMT-11
U-118 WRT lnc-GBMT-11
U-118 WRT lnc-GBMT-11
NTC lnc-GBMT-11
500 bp
200 bp
100 bp

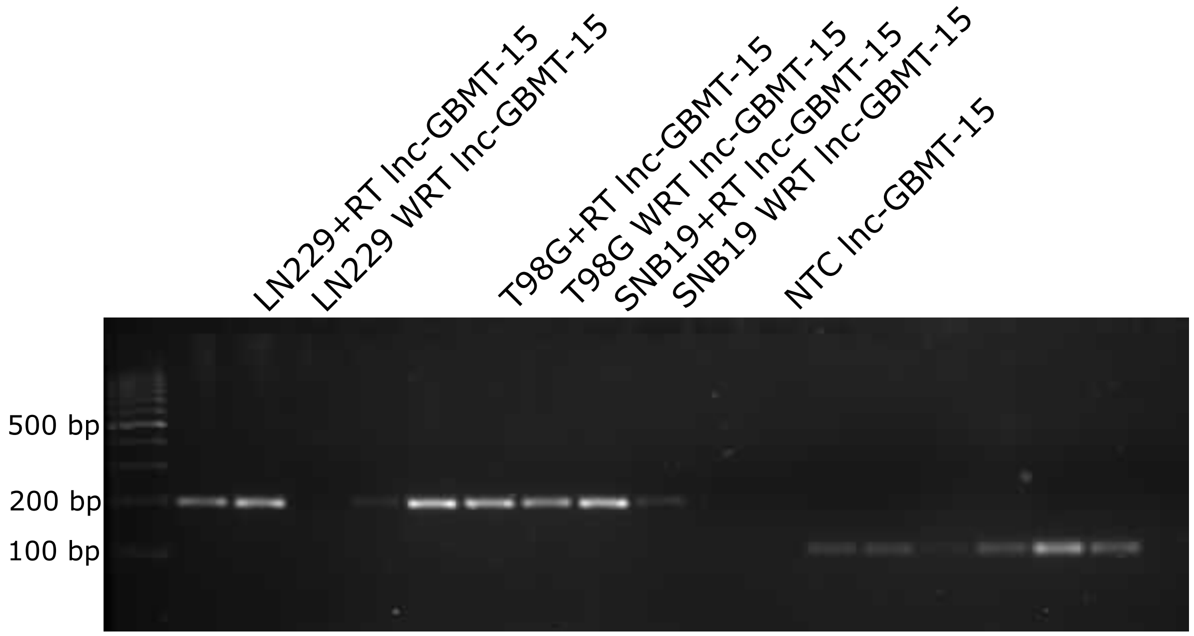
LN229+RT lnc-GBMT-15
LN229 WRT lnc-GBMT-15
T98G+RT lnc-GBMT-15
T98G WRT lnc-GBMT-15
SNB19+RT lnc-GBMT-15
SNB19 WRT lnc-GBMT-15
NTC lnc-GBMT-15
500 bp
200 bp
100 bp

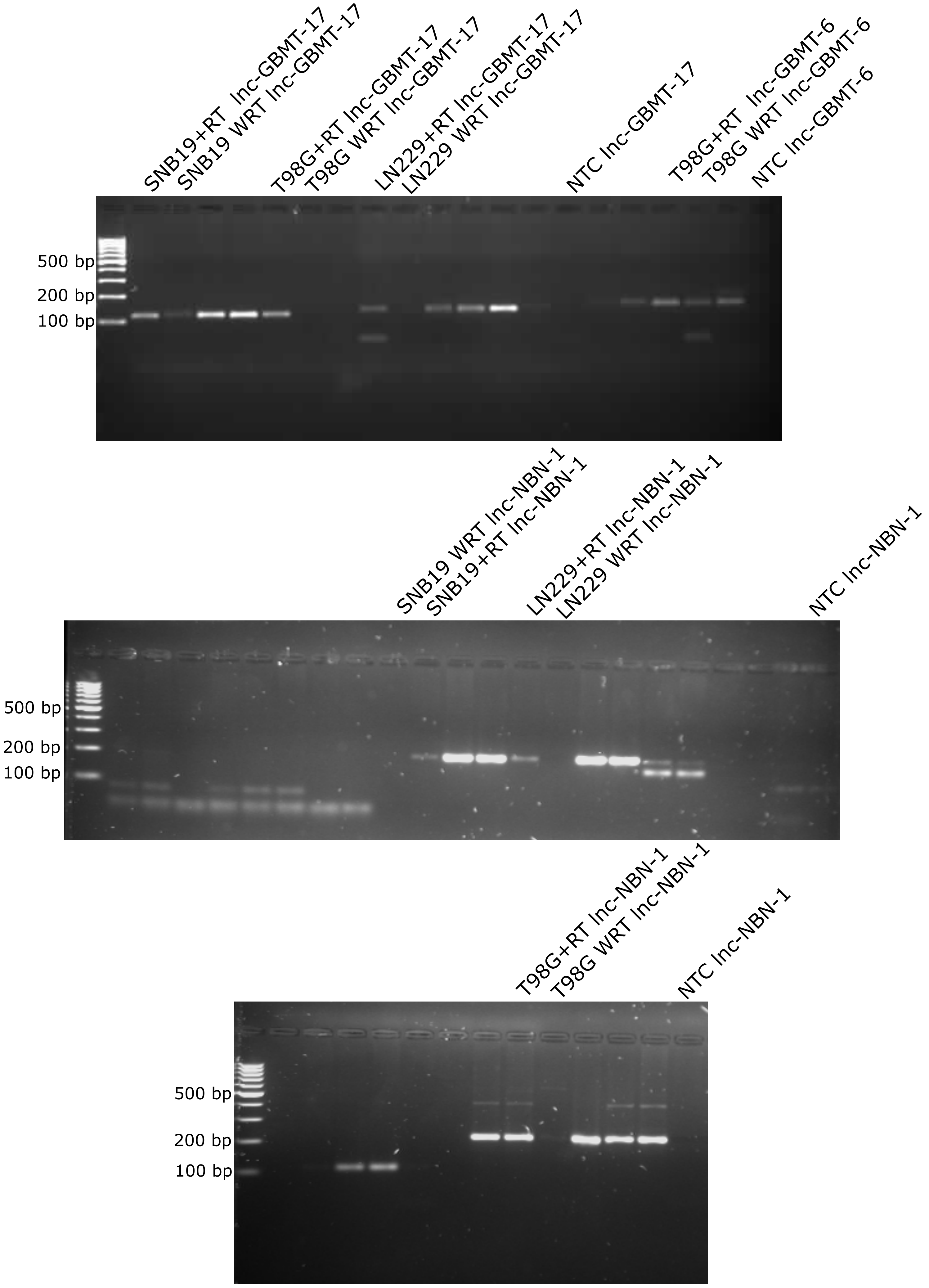

SNB19+RT lnc-GBMT-17
SNB19 WRT lnc-GBMT-17
T98G+RT lnc-GBMT-17
T98G WRT lnc-GBMT-17
LN229+RT lnc-GBMT-17
LN229 WRT lnc-GBMT-17
NTC lnc-GBMT-17
T98G+RT lnc-GBMT-6
T98G WRT lnc-GBMT-6
NTC lnc-GBMT-6
500 bp
200 bp
100 bp
SNB19 WRT lnc-NBN-1
SNB19+RT lnc-NBN-1
LN229+RT lnc-NBN-1
LN229 WRT lnc-NBN-1
NTC lnc-NBN-1
500 bp
200 bp
100 bp
T98G+RT lnc-NBN-1
T98G WRT lnc-NBN-1
NTC lnc-NBN-1
500 bp
200 bp
100 bp

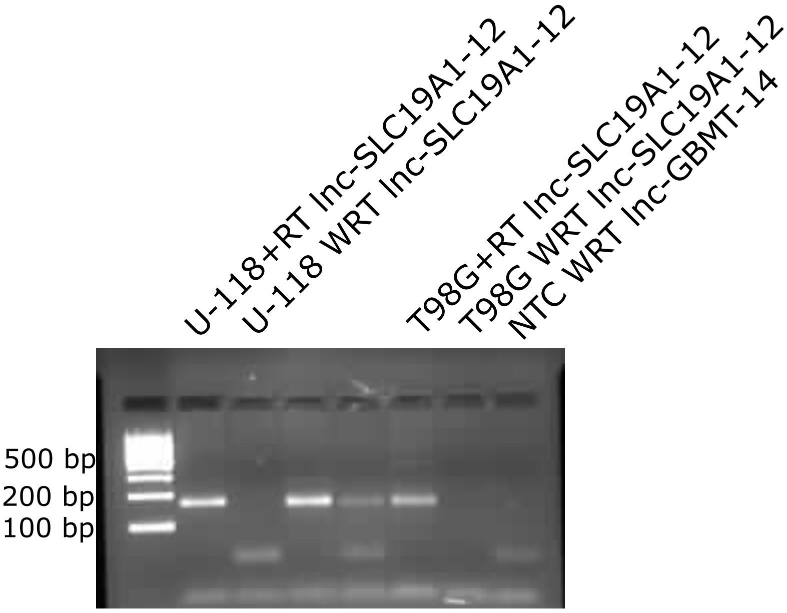
U-118+RT lnc-SLC19A1-12
U-118 WRT lnc-SLC19A1-12
T98G+RT lnc-SLC19A1-12
T98G WRT lnc-SLC19A1-12
NTC WRT lnc-GBMT-14
500 bp
200 bp
100 bp

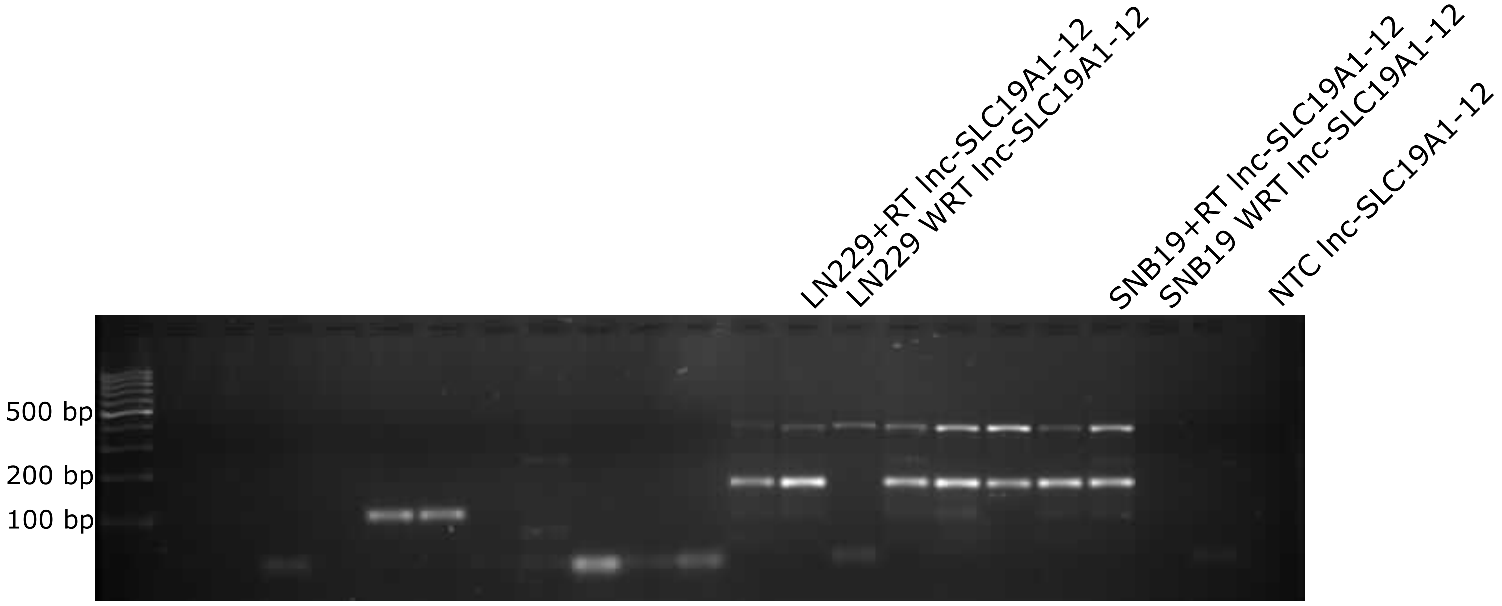
LN229+RT lnc-SLC19A1-12
LN229 WRT lnc-SLC19A1-12
SNB19+RT lnc-SLC19A1-12
SNB19 WRT lnc-SLC19A1-12
NTC lnc-SLC19A1-12
500 bp
200 bp
100 bp

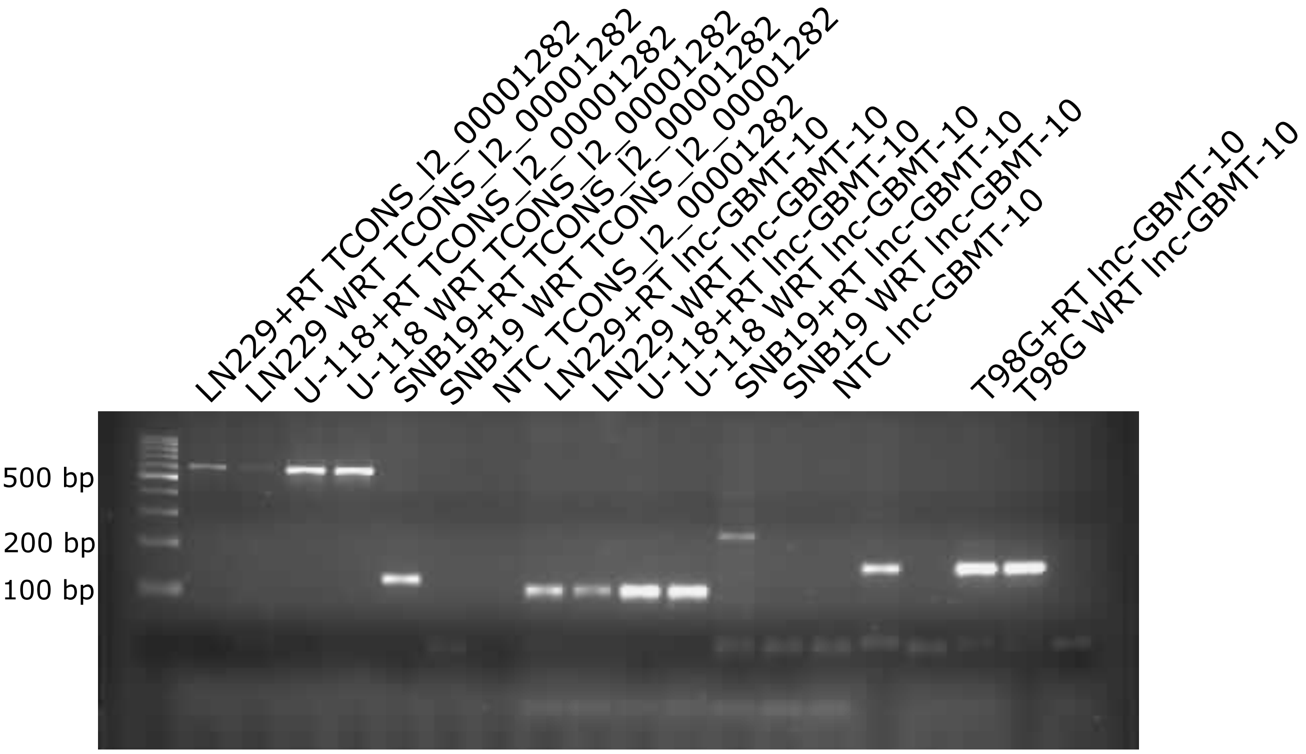
LN229+RT TCONS_l2_00001282
LN229 WRT TCONS_l2_00001282
U-118+RT TCONS_l2_00001282
U-118 WRT TCONS_l2_00001282
SNB19+RT TCONS_l2_00001282
SNB19 WRT TCONS_l2_00001282
NTC TCONS_l2_00001282
LN229+RT lnc-GBMT-10
LN229 WRT lnc-GBMT-10
U-118+RT lnc-GBMT-10
U-118 WRT lnc-GBMT-10
SNB19+RT lnc-GBMT-10
SNB19 WRT lnc-GBMT-10
NTC lnc-GBMT-10
T98G+RT lnc-GBMT-10
T98G WRT lnc-GBMT-10
500 bp
200 bp
100 bp

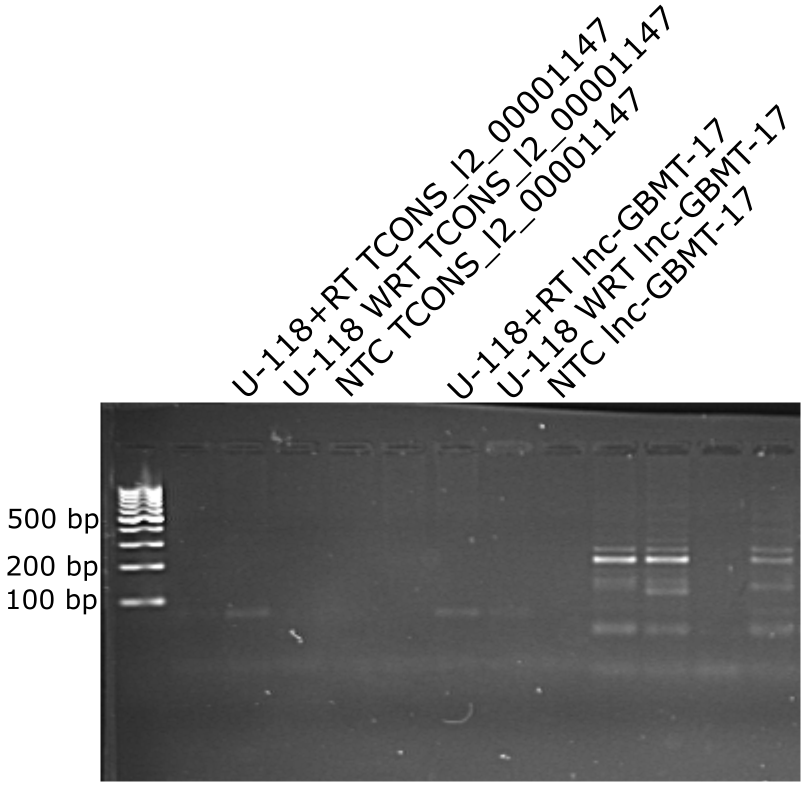

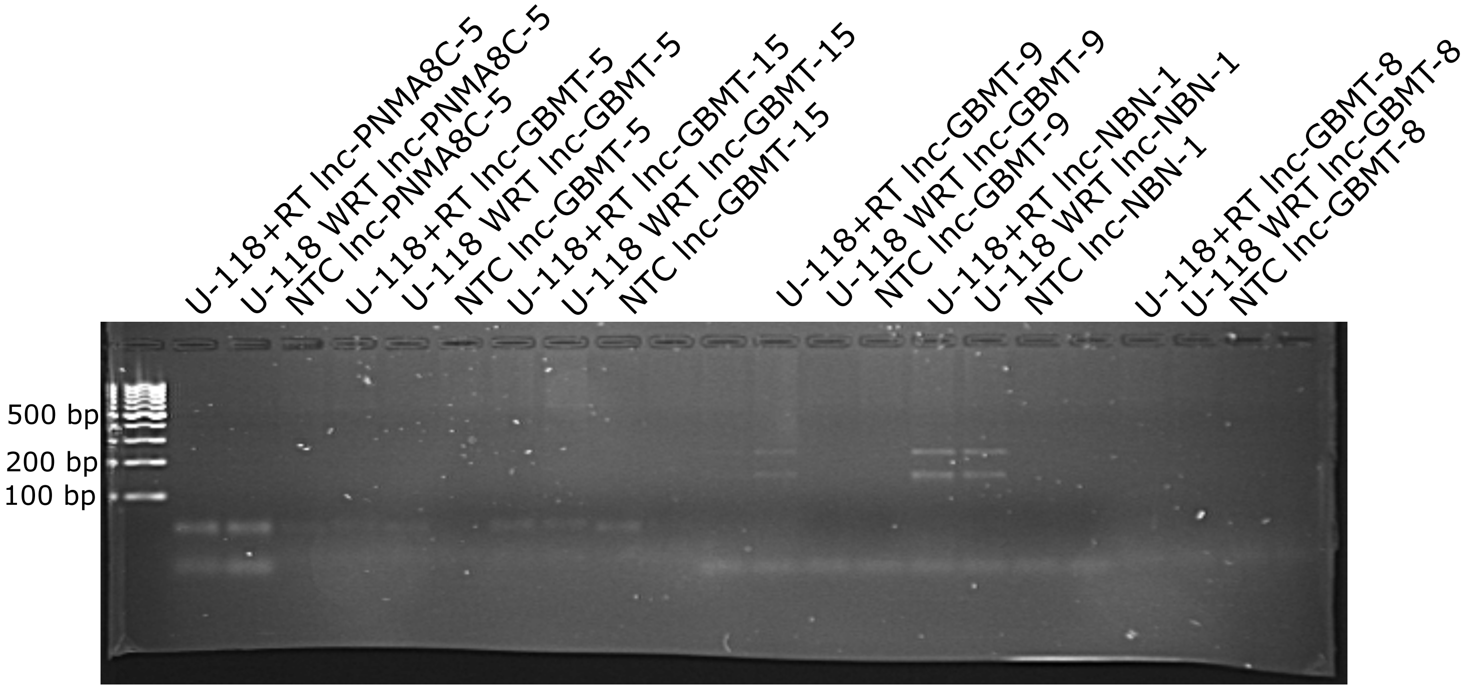

Supplementary Figure 2: a) Raw gel images for each lncRNA from each cell line. lncRNAs were considered to be expressed when a band was detected after the reverse transcription PCR (name of the cell line+RT) and there was no band when RT-PCR was done without reverse transcriptase before PCR (without reverse transcriptase, WRT). Ladder size in base pairs (bp) is on the left side of each figure. Summary of these results can be seen in the left side panel of Figure 1C.

A

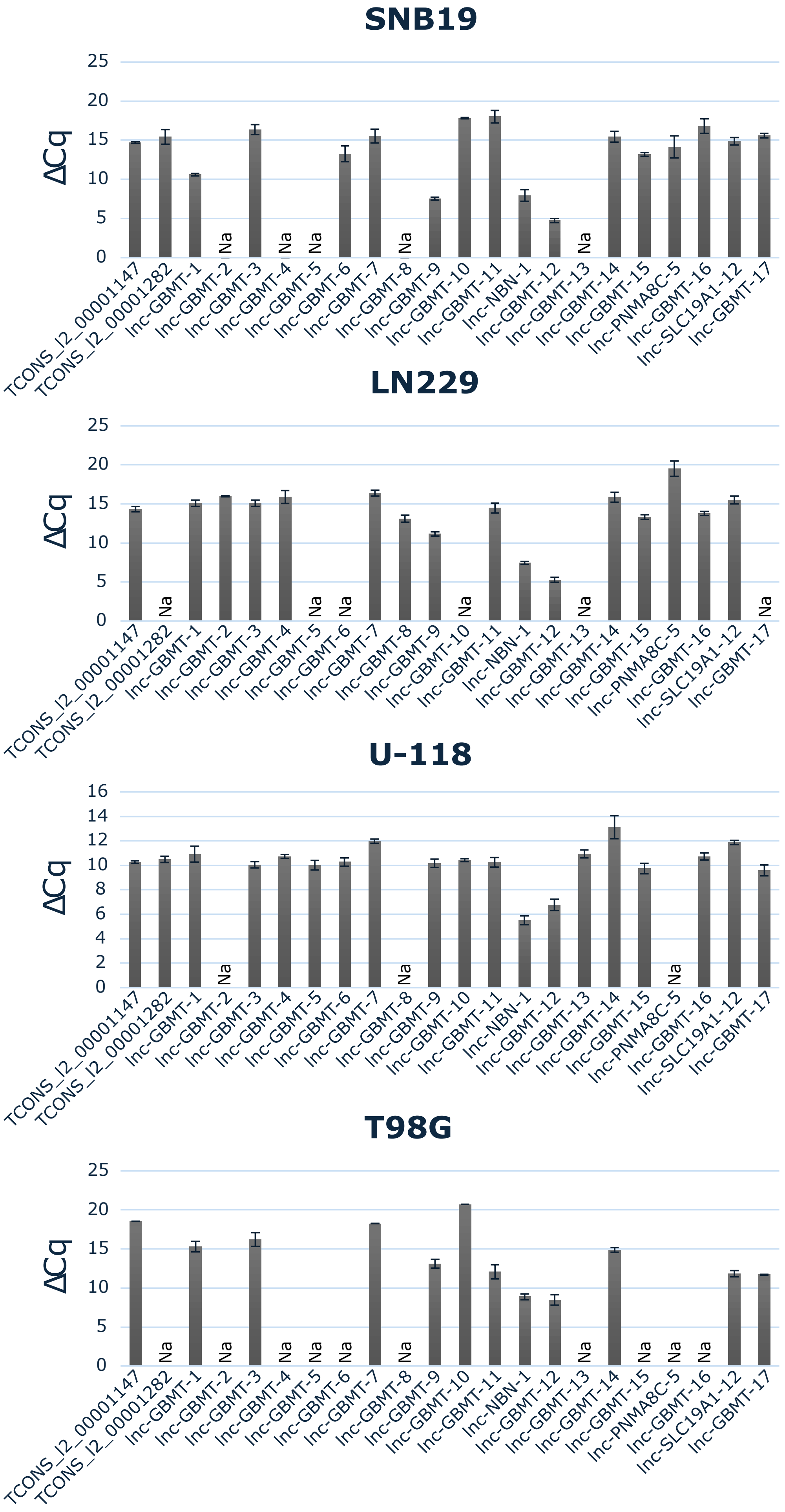

Supplementary Figure 3: a) Mean delta Cq values for each lncRNA measured in four different GBM cell lines. The results show the mean expression difference of the GAPDH to the given lncRNA. Lower value means higher expression. Standard deviation is presented with whiskers on top of each bar plot. The values represent measurement from three separate biological replicates each including three technical replicates.

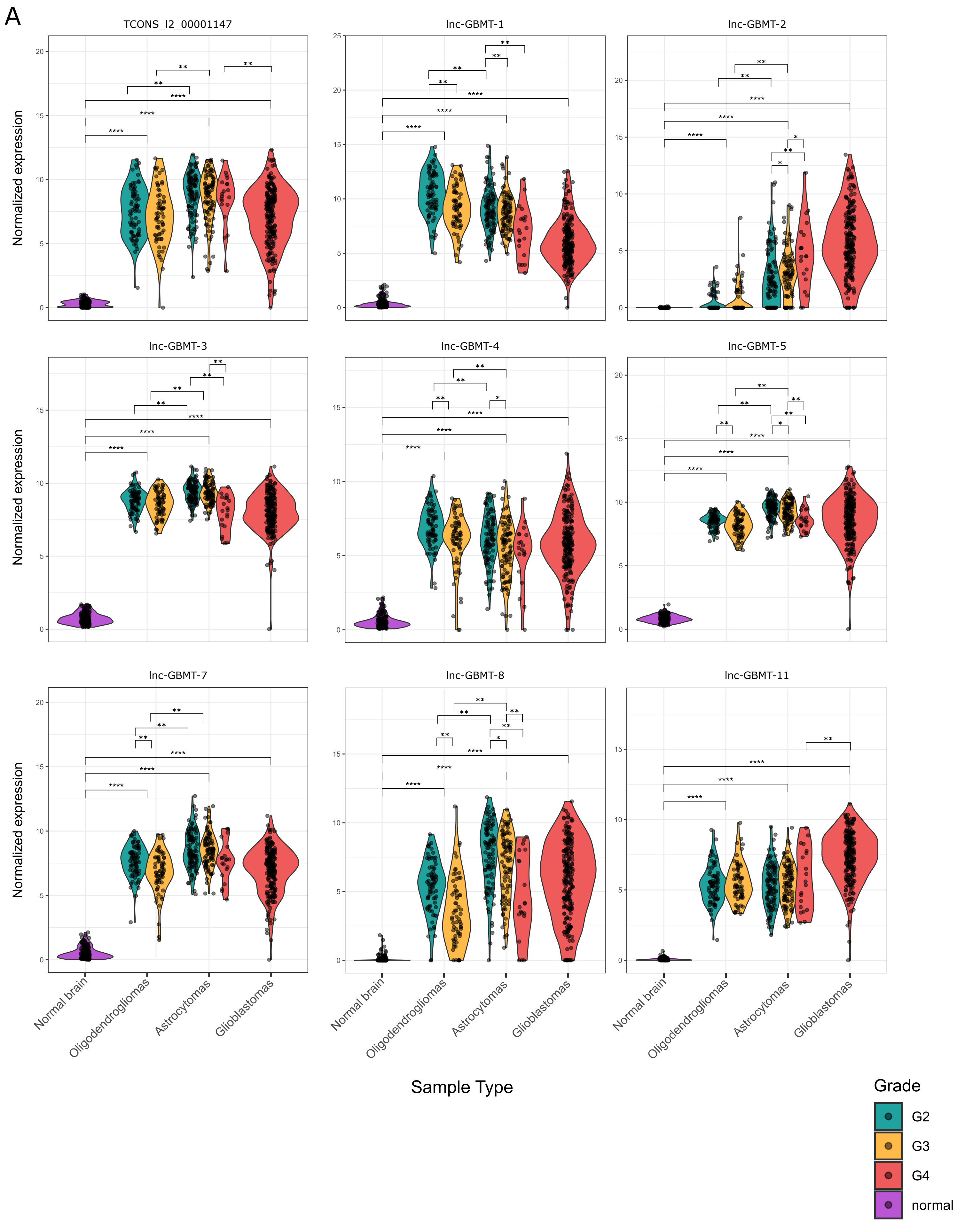

Supplementary Figure 4

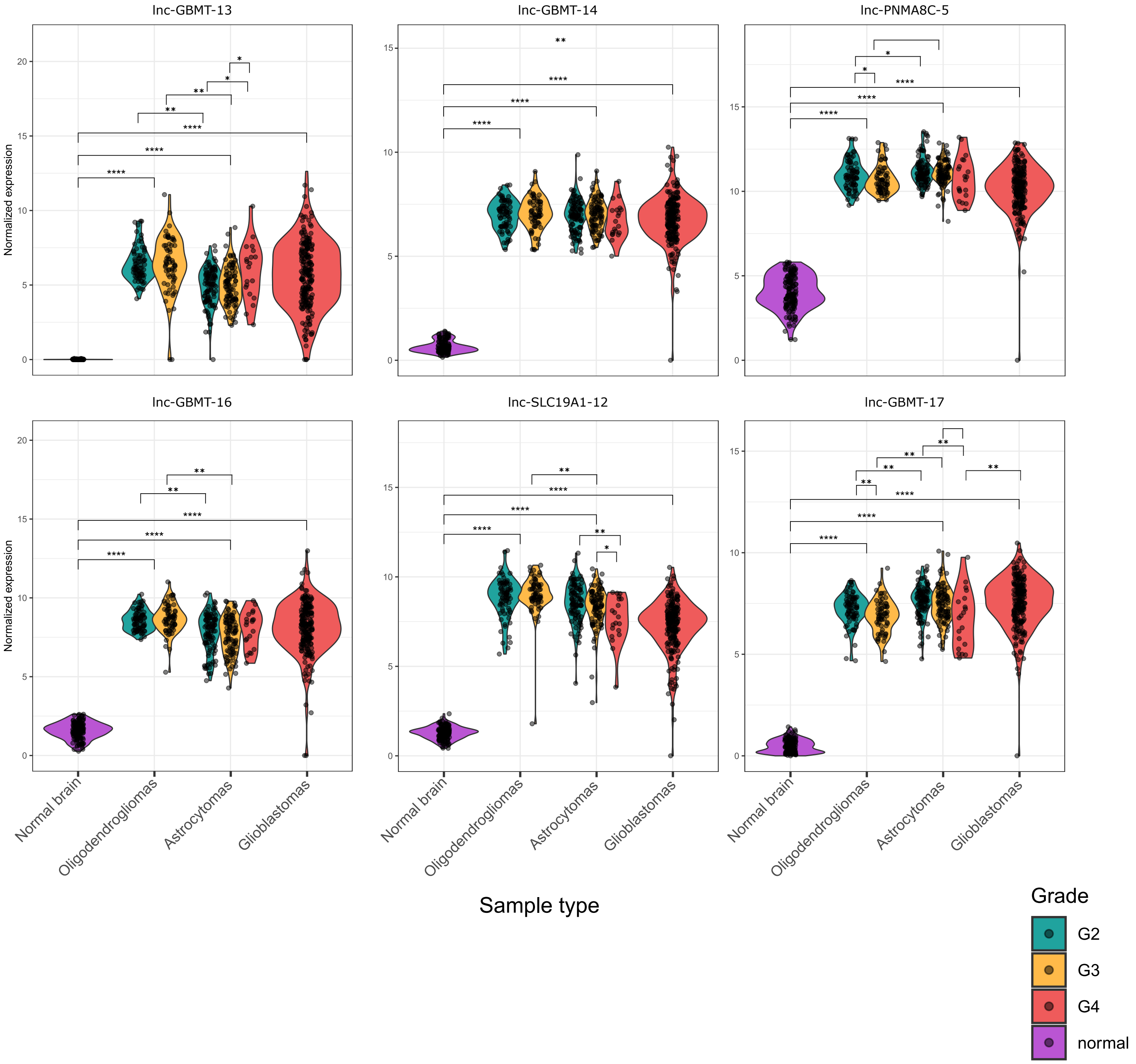

Supplementary Figure 4: a) Expression patterns of 15 different lncRNAs not shown in Figure 2 in grade 2–4 astrocytomas, grade 2–3 oligodendrogliomas, grade 4 GBMs, and normal brain. Expression differences were calculated between different grades within one tumor entity or between tumor entities representing the same tumor grade or between normal brain and tumor entities. *p < 0.05, **p < 0.01, ****p < 0.0001, Wilcoxon rank-sum test.

A

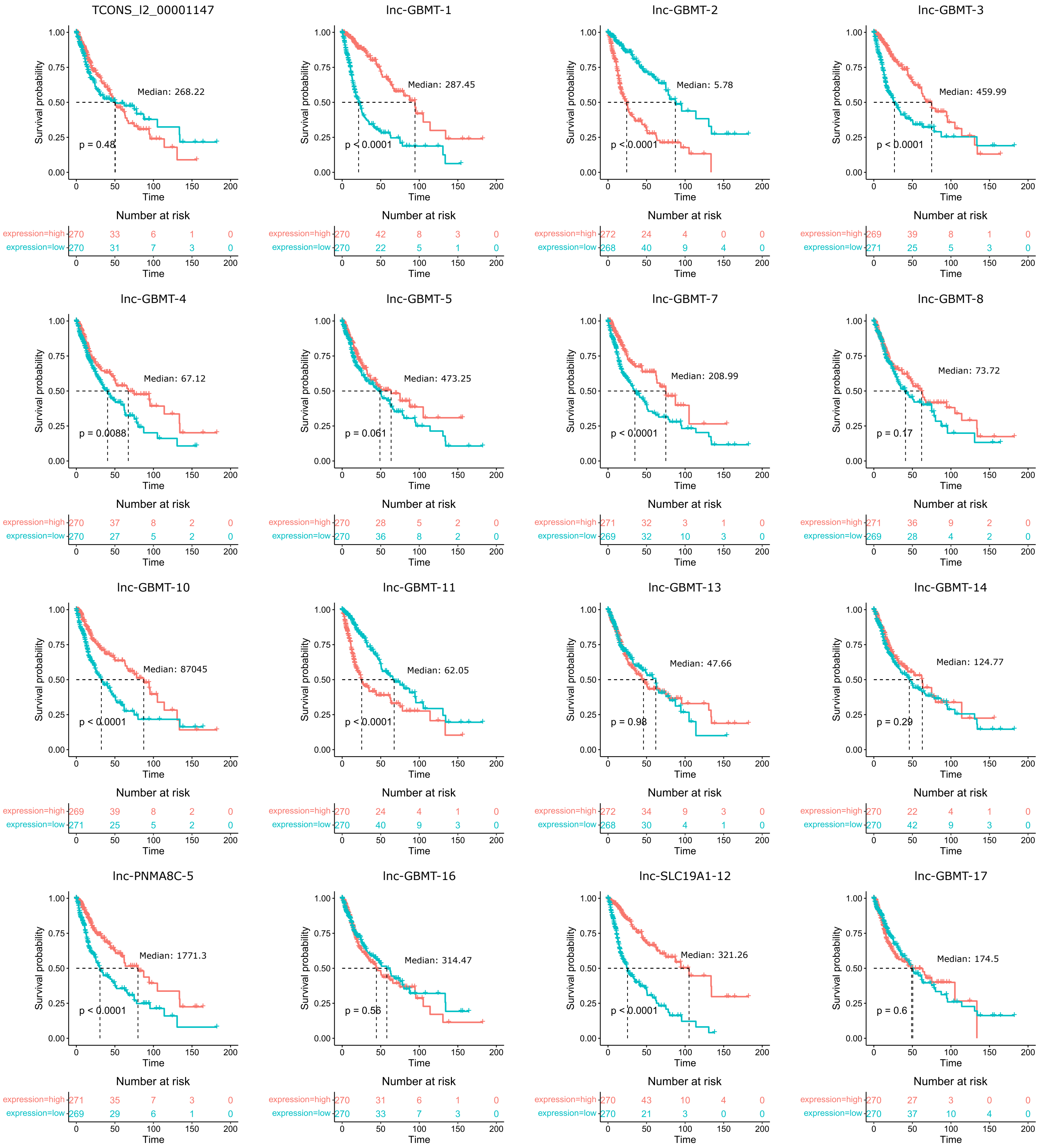

Supplementary Figure 5: a) Kaplan-Meier visualizations of the survival analysis in the whole diffuse glioma cohort for the lncRNAs not shown in Figure 2. Median expression was used as a cutoff for low/high lncRNA expression. Statistical significance was calculated with a log-rank test. b) Kaplan-Meier visualizations of the survival analysis in the IDH-mutant astrocytoma cohort. Median expression was used as a cutoff for low/high lncRNA expression. Statistical significance was calculated with a log-rank test. c) Kaplan-Meier visualizations of the survival analysis in the oligodendroglioma cohort. Median expression was used as a cutoff for low/high lncRNA expression. Statistical significance was calculated with a log-rank test. d) Kaplan-Meier visualizations of the survival analysis in the GBM cohort. Median expression was used as a cutoff for low/high lncRNA expression. Statistical significance was calculated with a log-rank test

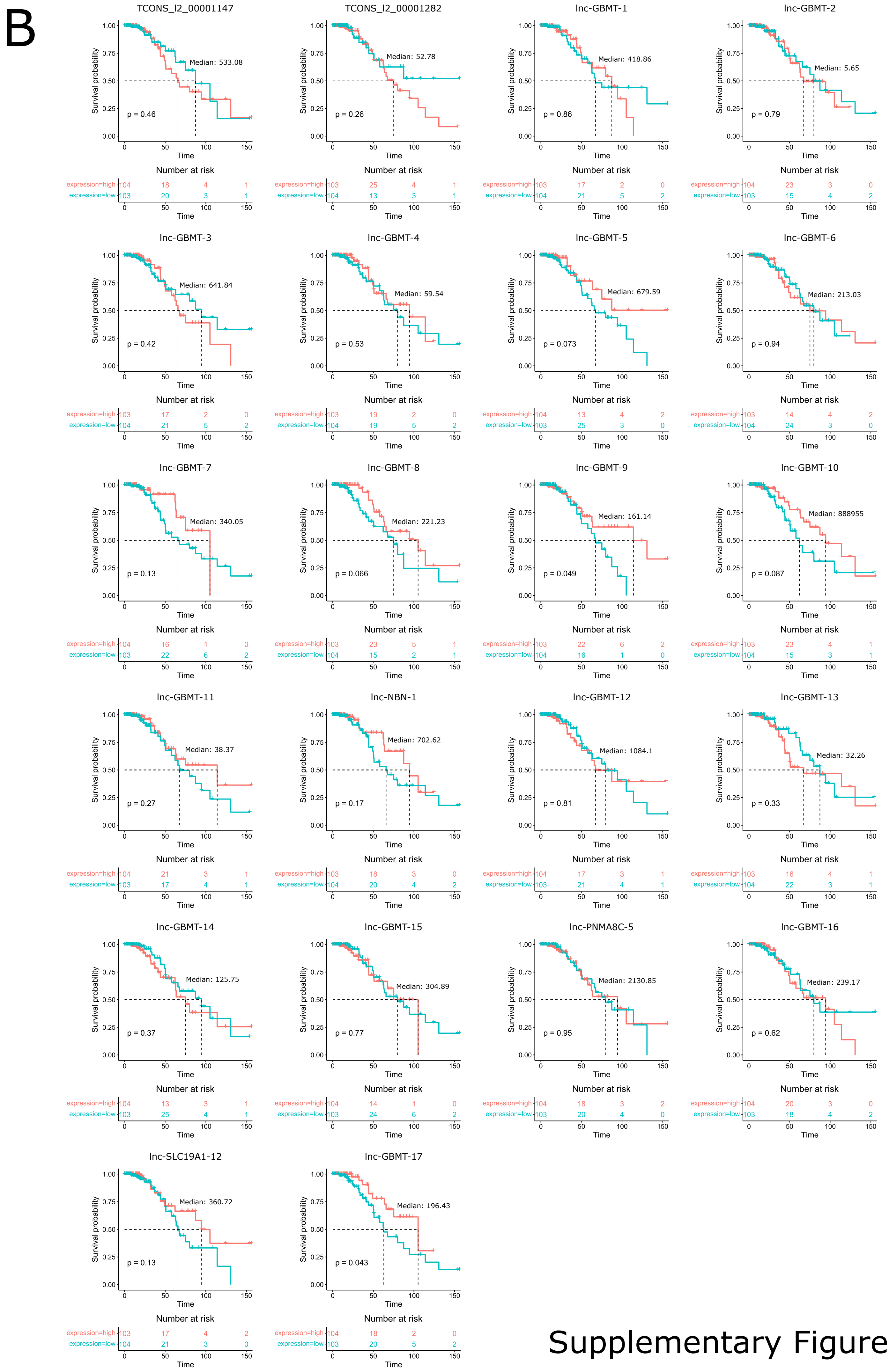

Supplementary Figure 5

C

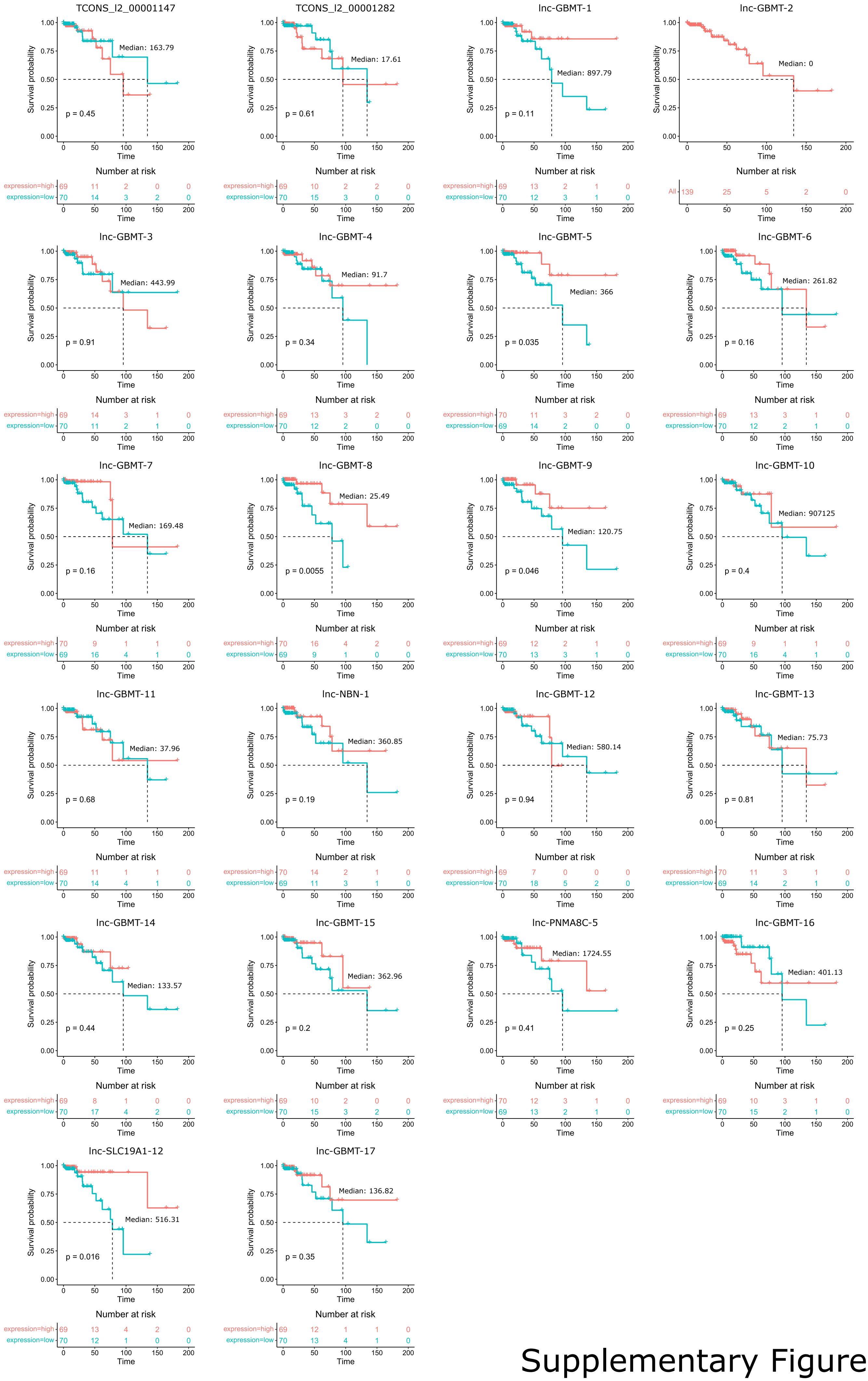

Supplementary Figure 5

D

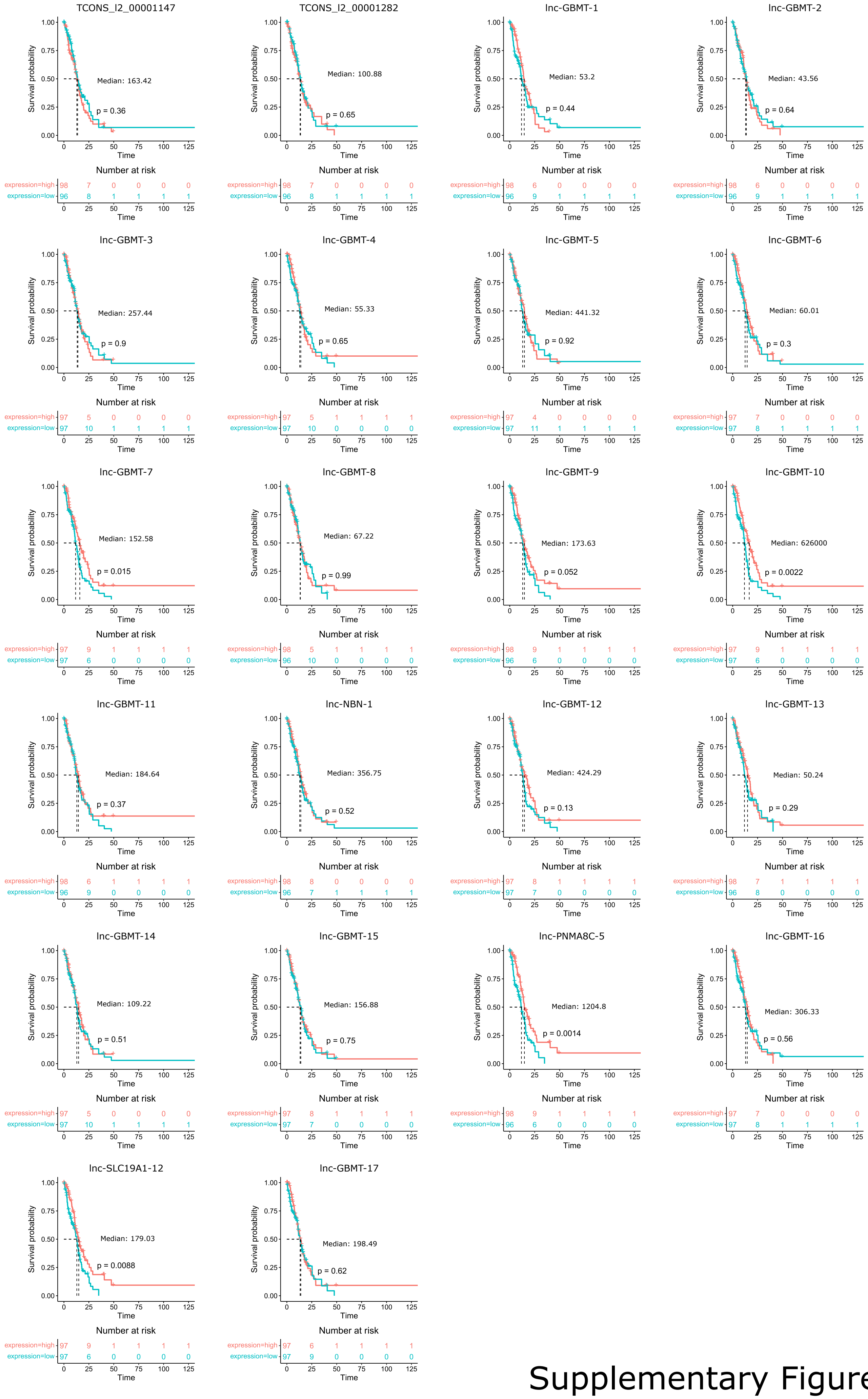

Supplementary Figure 5

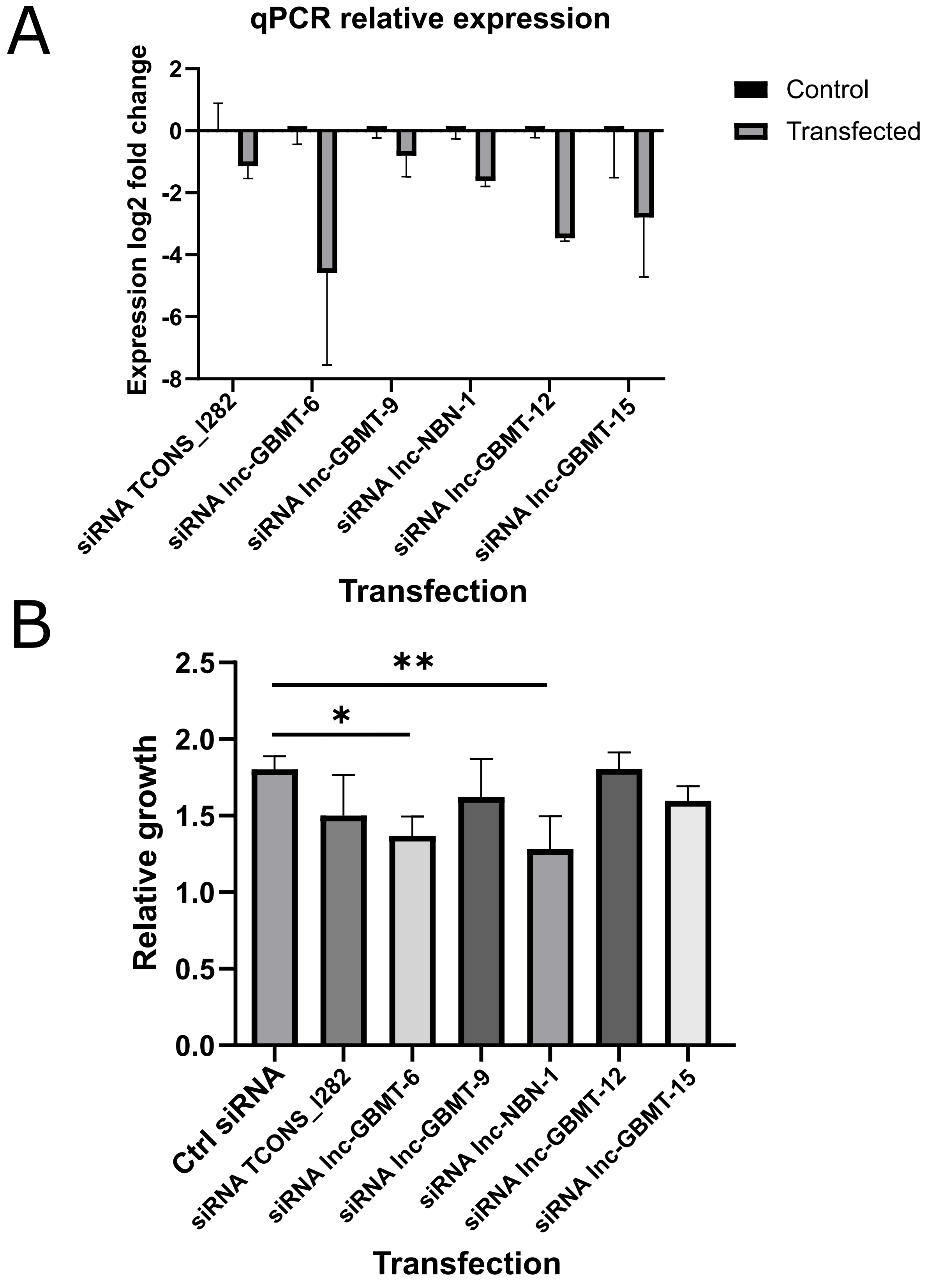

Supplementary Figure 6: a) Relative expression of six lncRNAs used for functional experiments after targeted or control siRNA transfection in the SNB19 cell line. All the lncRNAs presented reduced expression after siRNA transfection. The results show the mean expression difference together with the standard deviation from measurements representing three separate biological experiments (separate wells on a well plate), each with three technical replicates (separate measurements from one well). b) Alamar Blue assay showing reduced growth after the silencing of six selected lncRNAs. The figure shows the relative mean growth after five days from six biological replicates (separate wells on a well-plate) with the standard deviation in comparison to that on day zero. *$p < 0.05$, **$p < 0.01$, one-way ANOVA. From each biological replicate, six technical replicates (separate measurements from one well) were taken for the Alamar Blue assay. In this experiment where we analyzed all six transcripts, two (lnc-GBMT-6 and lnc-NBN-1) showed a significant decrease in growth. Reductions in cell growth after silencing of TCONS_l2_00001282, lnc-GBMT-6, or lnc-NBN-1 were observed after this experiment in at least two separate experiments for each lncRNA. One of these experiments is shown in Figure 3B.

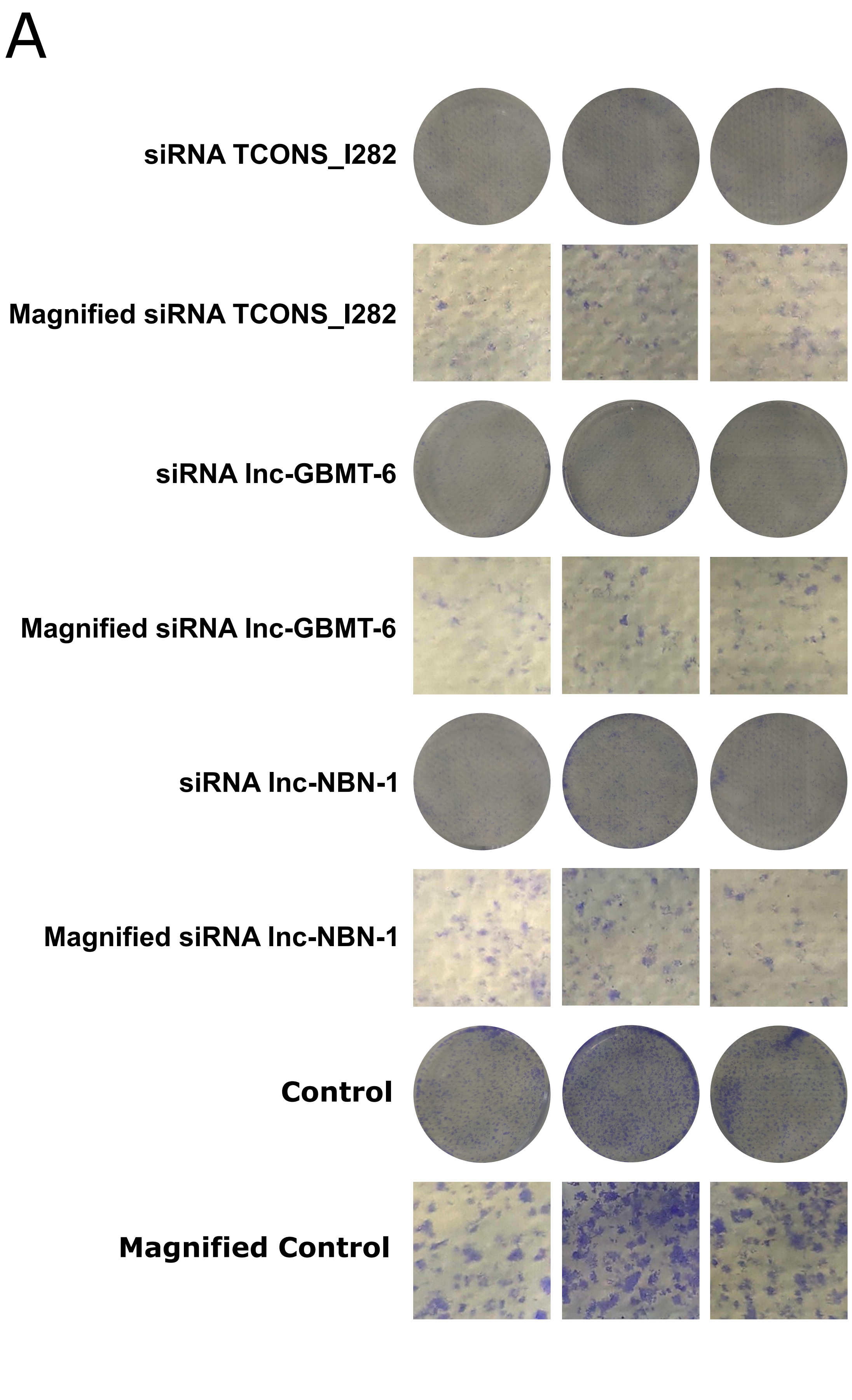

Supplementary Figure 7: a) Representative images and magnifications of center area for each of cell culture dishes after the clonogenic assay for TCONS_l2_00001282, lnc-GBMT-6, and lnc-NBN-1 lncRNAs that had growth effects in the Alamar Blue assay.

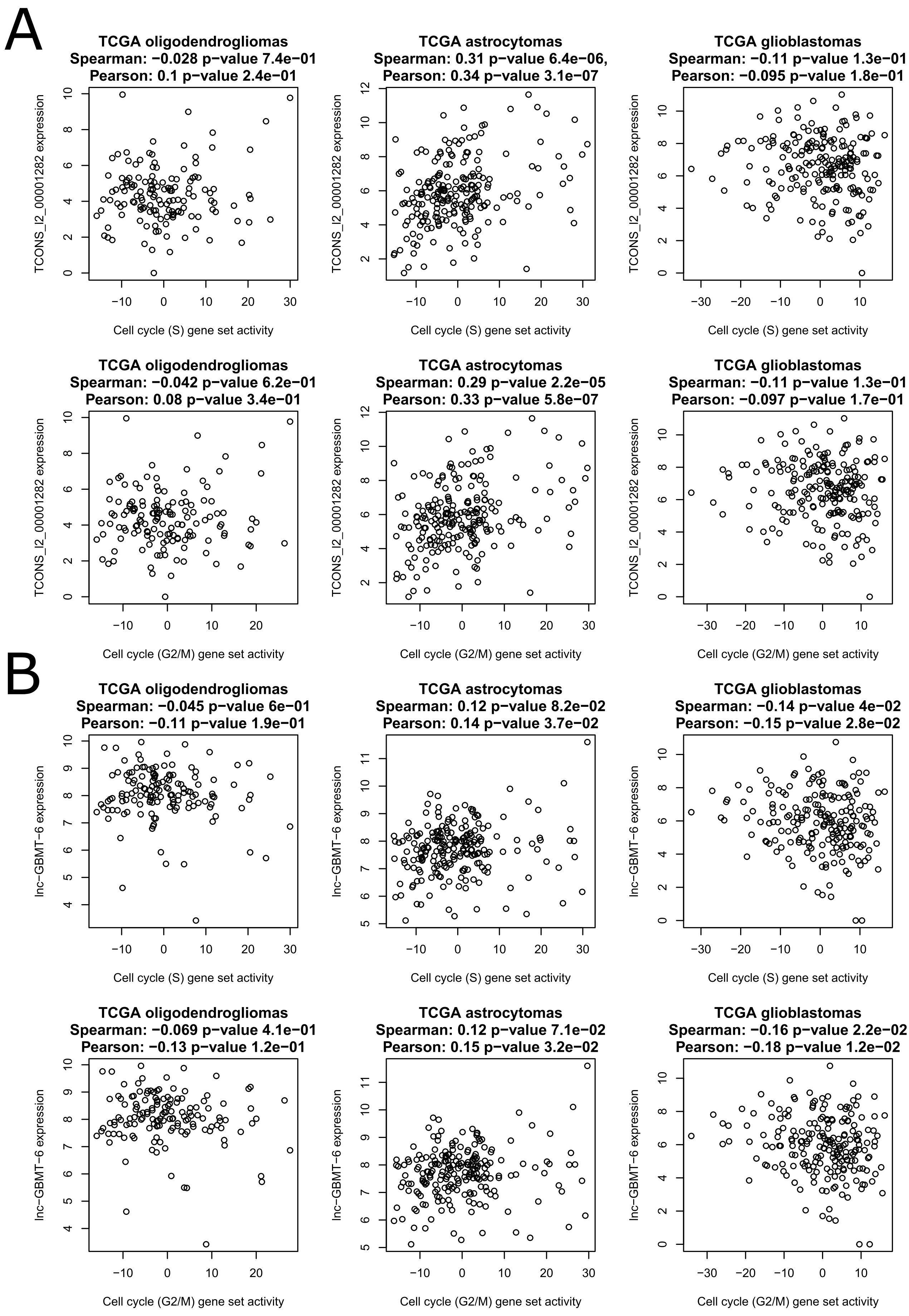
A
TCGA oligodendrogliomas
Spearman: −0.028 p-value 7.4e−01
Pearson: 0.1 p-value 2.4e−01
TCONS_l2_00001282 expression
Cell cycle (S) gene set activity
TCGA astrocytomas
Spearman: 0.31 p-value 6.4e−06,
Pearson: 0.34 p-value 3.1e−07
TCONS_l2_00001282 expression
Cell cycle (S) gene set activity
TCGA glioblastomas
Spearman: −0.11 p-value 1.3e−01
Pearson: −0.095 p-value 1.8e−01
TCONS_l2_00001282 expression
Cell cycle (S) gene set activity
TCGA oligodendrogliomas
Spearman: −0.042 p-value 6.2e−01
Pearson: 0.08 p-value 3.4e−01
TCONS_l2_00001282 expression
Cell cycle (G2/M) gene set activity
TCGA astrocytomas
Spearman: 0.29 p-value 2.2e−05
Pearson: 0.33 p-value 5.8e−07
TCONS_l2_00001282 expression
Cell cycle (G2/M) gene set activity
TCGA glioblastomas
Spearman: −0.11 p-value 1.3e−01
Pearson: −0.097 p-value 1.7e−01
TCONS_l2_00001282 expression
Cell cycle (G2/M) gene set activity
B
TCGA oligodendrogliomas
Spearman: −0.045 p-value 6e−01
Pearson: −0.11 p-value 1.9e−01
lnc−GBMT−6 expression
Cell cycle (S) gene set activity
TCGA astrocytomas
Spearman: 0.12 p-value 8.2e−02
Pearson: 0.14 p-value 3.7e−02
lnc−GBMT−6 expression
Cell cycle (S) gene set activity
TCGA glioblastomas
Spearman: −0.14 p-value 4e−02
Pearson: −0.15 p-value 2.8e−02
lnc−GBMT−6 expression
Cell cycle (S) gene set activity
TCGA oligodendrogliomas
Spearman: −0.069 p-value 4.1e−01
Pearson: −0.13 p-value 1.2e−01
lnc−GBMT−6 expression
Cell cycle (G2/M) gene set activity
TCGA astrocytomas
Spearman: 0.12 p-value 7.1e−02
Pearson: 0.15 p-value 3.2e−02
lnc−GBMT−6 expression
Cell cycle (G2/M) gene set activity
TCGA glioblastomas
Spearman: −0.16 p-value 2.2e−02
Pearson: −0.18 p-value 1.2e−02
lnc−GBMT−6 expression
Cell cycle (G2/M) gene set activity

C

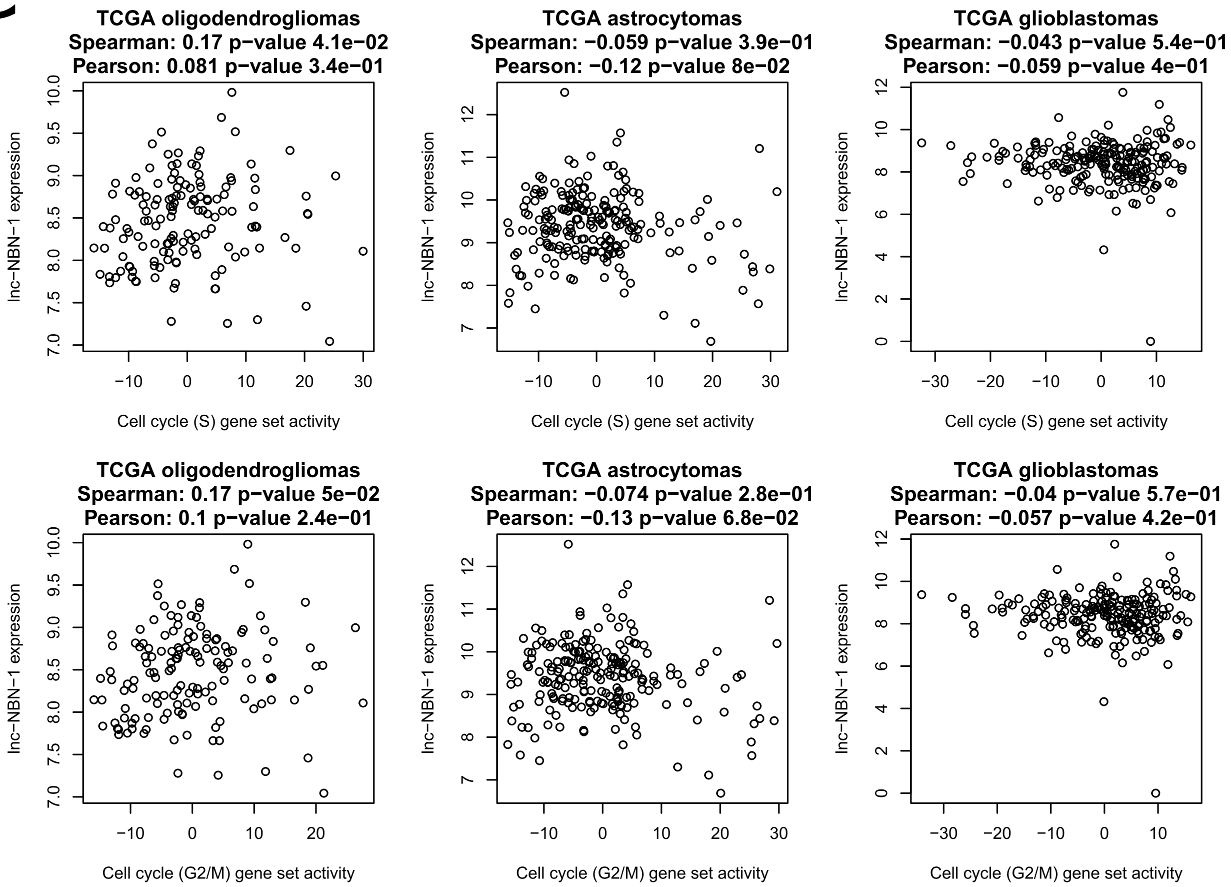

Supplementary Figure 8: a) Cell cycle gene set (S and G2/M) activities correlation with TCONS_l2_00001282 expression in TCGA diffuse gliomas. TCONS_l2_00001282 shows correlation in IDH-mutant astrocytomas. b) Same as in a) but with lnc-GBMT-6. lnc-GBMT-6 shows weak correlation in IDH-mutant astrocytomas. c) Same as in a) but with lnc-NBN-1

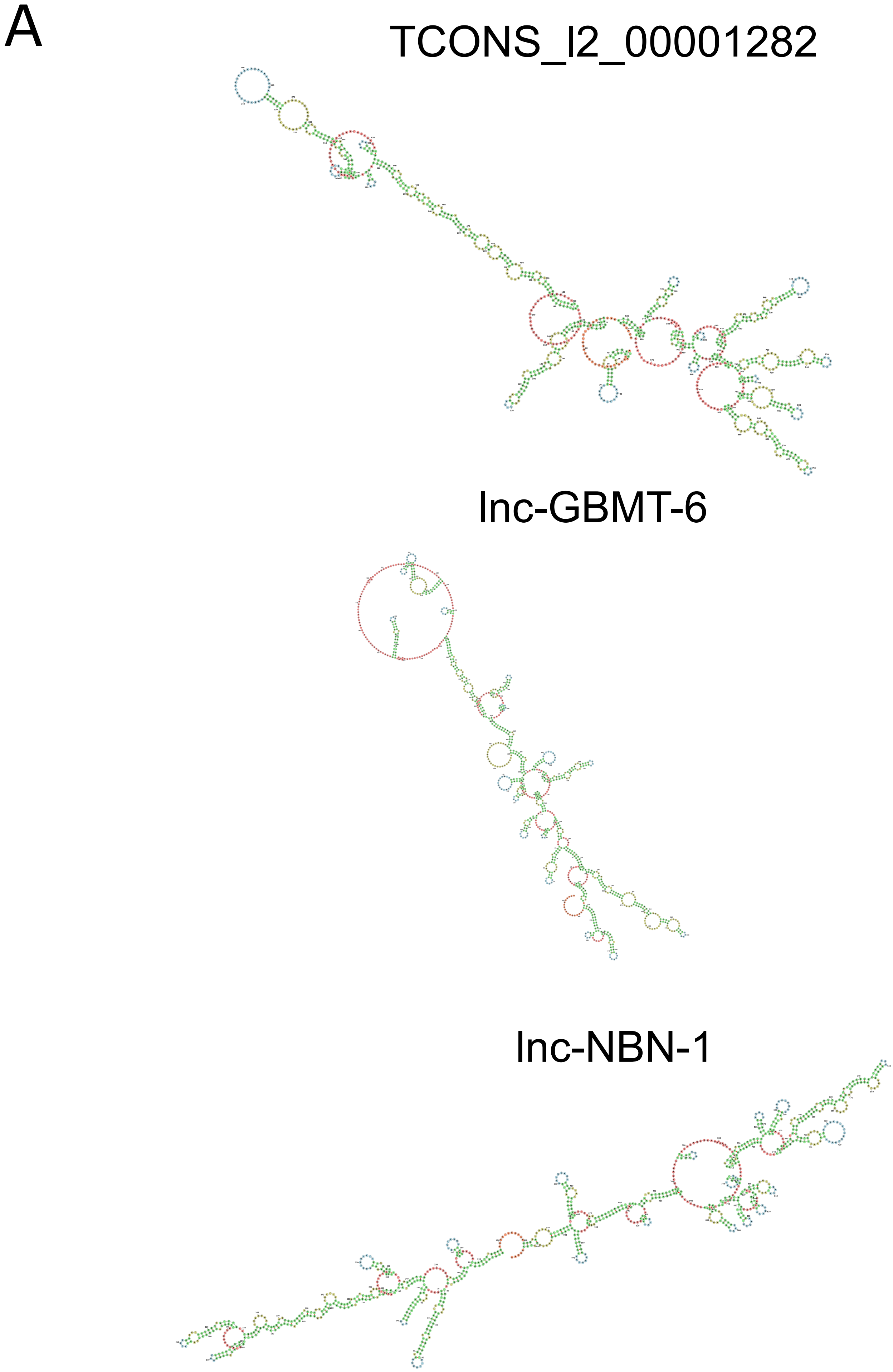

Supplementary Figure 9: a) Predicted secondary structures for TCONS_l2_00001282, lnc-GBMT-6, and lnc-NBN-1 lncRNAs. The secondary structures were derived using prediction tool developed by Sato et al.
